# The transcription factor DksA exerts opposing effects on cell division depending on the presence of ppGpp

**DOI:** 10.1128/mbio.02425-23

**Published:** 2023-10-26

**Authors:** Sarah E. Anderson, Stephen E. Vadia, Jane McKelvy, Petra Anne Levin

**Affiliations:** 1 Department of Biology, Washington University in St. Louis, Saint Louis, Missouri, USA; University of Nebraska Medical Center, Omaha, Nebraska, USA

**Keywords:** cell division, stringent response, transcription factors

## Abstract

Bacterial cell size is a multifactorial trait that is influenced by variables including nutritional availability and the timing of cell division. Prior work revealed a negative correlation between concentration of the alarmone (p)ppGpp (ppGpp) and cell length in *Escherichia coli*, suggesting that ppGpp may promote assembly of the division machinery (divisome) and cytokinesis in this organism. To clarify this counterintuitive connection between a starvation-induced stress response effector and cell proliferation, we undertook a systematic analysis of growth and division in *E. coli* cells defective in ppGpp synthesis and/or engineered to overproduce the alarmone. Our data indicate that ppGpp acts indirectly on divisome assembly through its role as a global mediator of transcription. Loss of either ppGpp (ppGpp^0^) or the ppGpp-associated transcription factor DksA led to increased average length, with ppGpp^0^ mutants also exhibiting a high frequency of extremely long filamentous cells. Using heat-sensitive division mutants and fluorescently labeled division proteins, we confirmed that ppGpp and DksA are cell division activators. We found that ppGpp and DksA regulate division through their effects on transcription, although the lack of known division genes or regulators in available transcriptomics data strongly suggests that this regulation is indirect. We also found that DksA inhibits division in ppGpp^0^ cells, contrary to its role in a wild-type background. We propose that the ability of ppGpp to switch DksA from a division inhibitor to a division activator helps tune cell length across different concentrations of ppGpp.

**IMPORTANCE:**

Cell division is a key step in the bacterial lifecycle that must be appropriately regulated to ensure survival. This work identifies the alarmone (p)ppGpp (ppGpp) as a general regulator of cell division, extending our understanding of the role of ppGpp beyond a signal for starvation and other stress. Even in nutrient-replete conditions, basal levels of ppGpp are essential for division to occur appropriately and for cell size to be maintained. This study establishes ppGpp as a “switch” that controls whether the transcription factor DksA behaves as a division activator or inhibitor. This unexpected finding enhances our understanding of the complex regulatory mechanisms employed by bacteria to coordinate division with diverse aspects of cell growth and stress response. Because division is an essential process, a better understanding of the mechanisms governing the assembly and activation of the division machinery could contribute to the development of novel therapeutics to treat bacterial infections.

## INTRODUCTION

Bacteria must integrate information about their external environments and nutritional states to grow and replicate successfully. The alarmone nucleotides pppGpp and ppGpp [(p)ppGpp, hereafter collectively referred to as ppGpp] are widely conserved throughout the bacterial kingdom and serve as an intracellular signal for starvation and other stresses. In the model bacterium *Escherichia coli*, ppGpp levels are controlled by two RelA/SpoT homologue (RSH) family proteins: the synthetase RelA and the bifunctional synthetase/hydrolase SpoT (1). During amino acid starvation, binding of stalled ribosomes to RelA stimulates synthesis of ppGpp, raising its concentration as much as 100-fold over baseline in a phenomenon known as the stringent response (2, 3). Starvation for fatty acids, phosphate, carbon, or iron leads to SpoT-dependent accumulation of ppGpp, either by stimulating SpoT’s ppGpp synthesis activity (fatty acids, phosphate) or by inhibiting its ppGpp degradation ability (carbon) (4–6). Accumulation of ppGpp to high levels results in a reduction in overall biosynthesis, with exceptions for proteins required for adaptation to stressful conditions (1). ppGpp is also present at basal levels during balanced growth in nutrient-replete conditions (7). Basal ppGpp is proposed to play a role in homeostasis (3), but a full understanding of the role of this molecule during balanced growth remains elusive.

In *E. coli* and other gamma-proteobacteria, ppGpp exerts its effects via transcriptional and post-translational mechanisms. As a transcriptional regulator, ppGpp binds to RNA polymerase (RNAP) at one of two equal-affinity sites, dubbed Sites 1 and 2 (8–10). Binding of ppGpp to RNAP during the stringent response leads to up- or downregulation of over 700 genes (11). Site 1 is comprised of RNAP subunits ω and β’ (9), while Site 2 is formed at the interface between RNAP and the transcription factor DksA (8). Unlike most transcription factors, DksA binds directly to RNAP to both activate and inhibit transcription (12, 13). DksA can bind RNAP with or without ppGpp, but binding of ppGpp at Site 2 leads to conformational changes in both DksA and RNAP that increase or decrease transcription, depending on the promoter (8, 13–15). ppGpp cannot bind to Site 2 without DksA, and DksA and ppGpp do not appear to bind each other without RNAP (8). Usually, DksA and ppGpp work cooperatively to influence transcription; however, there are examples of ppGpp and DksA exerting independent or even opposing transcriptional and phenotypic effects (16–21). In addition to this transcriptional pathway, ppGpp also serves as a post-translational regulator by directly binding to at least 50 proteins to modulate their activities (3, 22–24). Together, these two pathways allow ppGpp to modulate cell physiology and metabolism in response to environmental conditions and intracellular stress. Notably, although commonly thought of as a stress-related alarmone, ppGpp is also important for homeostatic regulation during steady-state growth, and defects in ppGpp synthesis lead to dysregulation of multiple pathways (7, 16, 19, 25–27).

To ensure survival, information about environmental conditions must be appropriately coordinated with the cell cycle, including the timing and frequency of cell division. Cell division is mediated by the divisome, a multiprotein complex (10 essential core proteins in *E. coli*) that guides the placement and synthesis of septal peptidoglycan at midcell (28). During growth, the members of the divisome assemble hierarchically at midcell. In *E. coli*, divisome assembly begins with the recruitment of the cytoplasmic tubulin homolog FtsZ to the future site of division. FtsZ serves as a dynamic scaffold for the rest of the complex. Divisome assembly terminates with FtsN (28). Divisome assembly is tightly controlled, with FtsZ serving as a major target of regulation during both balanced growth and stress (29–32). However, other components of the divisome can also be affected by regulation or environmental conditions; for example, FtsN is influenced by environmental pH (33). Regardless of mechanism, defects in division lead to increased cell length, while activation of division produces shorter cells.

Unsurprisingly, most components of the *E. coli* divisome are essential. In lieu of deletion mutations, conditional heat-sensitive alleles of division genes are extremely valuable for the study of divisome assembly and activation. Heat-sensitive mutations have been isolated in genes including *ftsZ*, *ftsA*, *ftsQ*, *ftsK*, and *ftsI* (34–37). When grown on lysogeny broth (LB) without salt (LBNS), heat-sensitive mutants *ftsZ84*, *ftsA12*, and *ftsQ1* support growth at 30°C but exhibit little to no growth at 37^o^ or 42°C (33, 38). Mutants *ftsI23* and *ftsK44* have milder phenotypes, permitting growth at temperatures up to 37°C, but exhibiting reduced growth at 42°C on LBNS (33). Notably, the heat sensitivity of many of these alleles can be suppressed by gain-of-function mutations in or overexpression of other cell division genes (38–40), or by changes in the environment that promote divisome assembly (33). For example, the gain-of-function mutation ftsA* can suppress heat sensitivity of multiple conditional division mutants, including *ftsK44* and *ftsQ1* (41).

Significant data argue for a positive relationship between ppGpp and division. Initial evidence for divisome activation by ppGpp was reported in 1998 by Powell and Court, who found that ppGpp overproduction increased survival of a heat-sensitive *ftsZ* mutant (*ftsZ84*) at restrictive temperatures (42). In addition, there is a negative correlation between ppGpp levels and cell length and, to a more modest degree, cell width (19, 26, 43, 44). Cells unable to synthesize ppGpp (referred to as ppGpp^0^) or defective in *dksA* sometimes filament (19, 26). ppGpp^0^ cells are also more sensitive to increased production of SulA, an inhibitor of FtsZ assembly (27), suggesting that even basal levels of ppGpp have a positive impact on cell division. Both ppGpp^0^ cells and cells overexpressing ppGpp grow slowly despite their dramatic differences in length, strongly suggesting that ppGpp does not control the size through its effects on growth rate (43). Modest increases in ppGpp cause resistance to mecillinam, an antibiotic targeting PBP2, the transpeptidase associated with the cell elongation machinery (elongasome) (45–47). Increased divisome activity can compensate for elongasome defects (48), suggesting that ppGpp causes mecillinam resistance by stimulating the divisome.

Transcriptomics data argue against a model in which ppGpp regulates expression of *ftsZ* or any other division genes (11, 16, 45, 49, 50). Increases in ppGpp do not impact FtsZ protein levels (45), nor do FtsZ or other division proteins appear to be direct binding partners of ppGpp (22, 24) (J.D. Wang, personal communication). While the work of Powell and Court suggests that increases in ppGpp suppress the heat sensitivity of *ftsZ84* by increasing FtsZ84 production, they normalized FtsZ84 levels to levels of another protein NusA, which is negatively regulated by ppGpp (11, 16, 42, 50). This likely caused FtsZ84 levels to appear artificially elevated in their ppGpp-overproducing strain. A more recent study reported a less than twofold decrease in FtsZ levels normalized to OD_600_ in a ppGpp^0^ strain (27). The mechanism underlying this apparent decrease remains unclear, as it does not align with transcriptional data available for the ppGpp^0^ strain.

Many open questions remain about the mechanism by which ppGpp affects cell size and division. In particular, the contribution of basal ppGpp to cell size has not been carefully studied. To our knowledge, the frequency of filamentation by ppGpp^0^ cells has never been quantified. Similarly, the effects of ppGpp on divisome assembly or on the survival of heat-sensitive division mutants other than *ftsZ84* have not been examined.

To gain insight into the mechanism by which ppGpp regulates cell division, we undertook a comprehensive analysis of the effect of ppGpp on divisome assembly and activation. We found that ppGpp is a positive regulator of divisome activity and assembly. Genetic interactions between ppGpp and *dksA* suggest that DksA exhibits opposing effects on division in WT and ppGpp^0^ cells during balanced growth. Overall, this work suggests a nuanced mechanism allowing cell length to be adjusted to different concentrations of ppGpp.

## RESULTS

### The impact of excess ppGpp on cell division is mediated through DksA

While previous work indicates that modest increases in ppGpp positively impact cell division in *E. coli* (42, 43), these studies did not determine the extent of this effect beyond suppression of *ftsZ84*, nor did they determine if ppGpp’s binding partner, DksA, was required. To address these gaps, we assessed the impact of excess ppGpp on *E. coli* cell size and division in the presence and absence of DksA. To increase ppGpp levels, we took advantage of a plasmid (*prelA*), which encodes *relA* under an IPTG-inducible promoter (51, 52) (Table 1; Table S1). A control plasmid, *prelA’* encodes an inactive *relA* allele, which is incapable of causing ppGpp accumulation in MG1655, under the same promoter (51) (Table 1; Table S1).

**TABLE 1:** Plasmids used in this study^*a*^

Plasmid	Gene encoded/description	Promoter(s)	Backbone	Citations
*prelA*	Full-length *relA*	P*_tac_*	*pMS119EH* (ColE1 origin)	(51, 53), this work
*prelA’*	Non-functional truncated *relA*			
*prelA-EV*	Empty vector (EV) control for *relA* plasmids.			
*pINIIIA* (EV)	EV control for *dksA* plasmids	P*_lpp_*, P*_lac_*	*pINIIIA* (high copy)	(8, 54–58)
*pdksA*	Wild-type *dksA*			
*pdksA_K98A_*	*dksA* allele defective for ppGpp binding			
*pdksA_N88I_*	Gain-of-function “super” *dksA*			
*pdksA_R91A_*	*dksA* allele deficient in RNAP binding			
*pdksA_D71N/D74N_*	*dksA* allele defective for transcriptional regulation			
*pftsQAZ*	*ftsQAZ*	Native promoters	*pGB2* (low copy)	(59), this work
*pftsZ*	*ftsZ*			
*pftsQA*	*ftsQA*			
*pftsN*	*gfp-ftsN* fusion	P*_lac_*	*pDR107A* (ColE1 origin)	(60, 61)

^*a*^ The gene(s) of interest encoded by each plasmid, the promoter(s) used to express them, and relevant information about each plasmid backbone are shown. Additional information about these plasmids, including their original published names and other encoded features, is available in Table S1.

**Fig 1:**
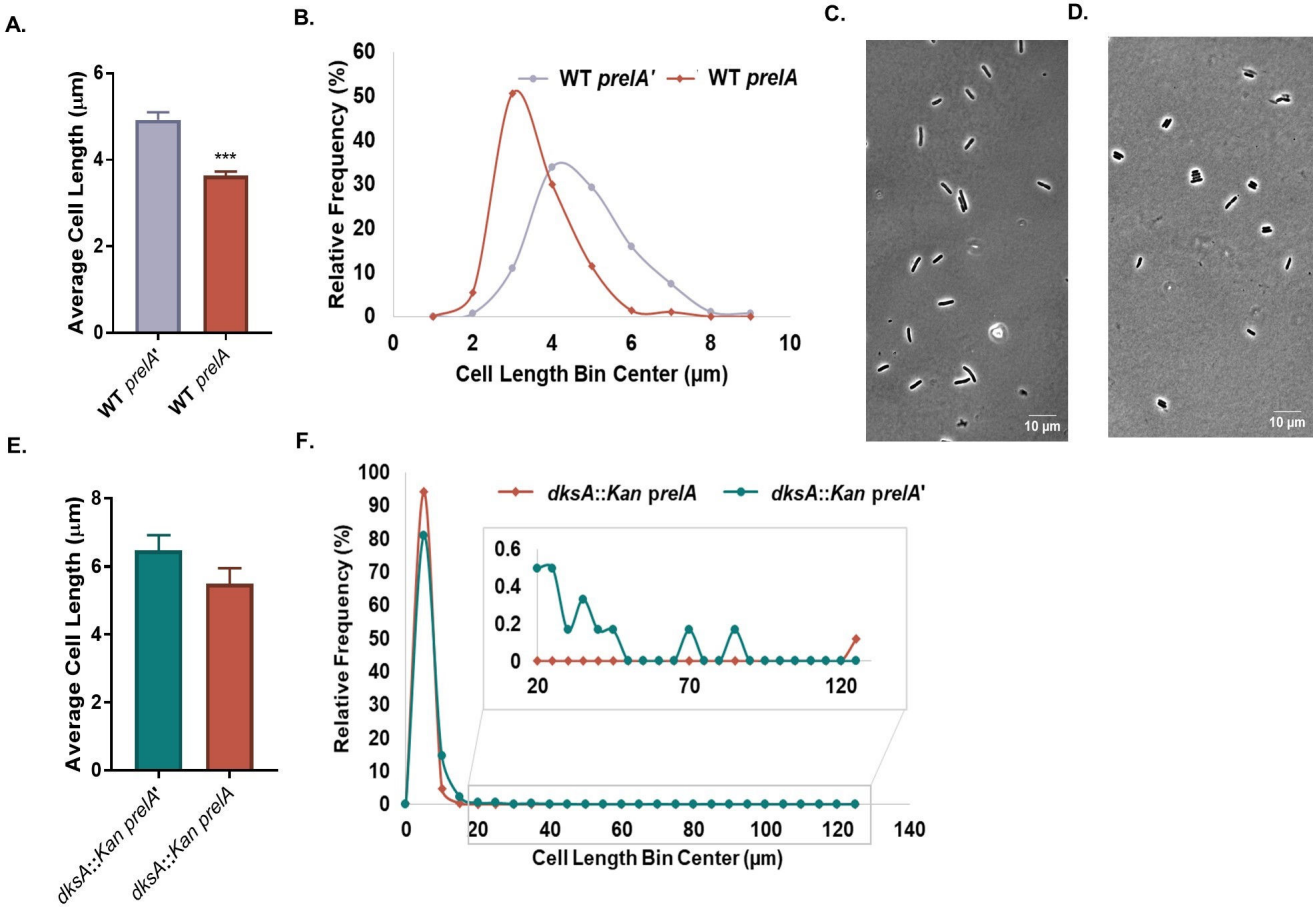
Excess ppGpp controls cell length through DksA. (A) WT cells overexpressing *relA* (*prelA*) are shorter than cells overexpressing a truncated non-functional *relA* (*prelA’*). Data represent averages and standard deviations (SDs) of three biological replicates (***P ≤ 0.001, two-tailed *t*-test). (B) Frequency distribution of lengths of individual cells expressing *prelA* or *prelA’*. *N* > 200 cells from a representative biological replicate (bin width = 1 µm). (C, D) Representative phase contrast micrographs of WT *prelA’* (**C**) and WT *prelA* (**D**) cells. (E) Cell length of *dksA::Kan* cells expressing *prelA* or *prelA’*. Data represent averages and SDs of three independent replicates (difference not significant by two-tailed *t*-test). (F) Frequency distribution of individual cell lengths for *dksA::Kan* cells overexpressing *prelA* or *prelA’*. *N* > 600 cells from a single representative experiment (bin width = 5 µm).

As a first step, we assessed the impact of *prelA* on cell size in the presence and absence of DksA. For these experiments, we sampled cells at exponential phase (OD_600_ = 0.1), fixed, imaged, and measured for cell size using MicrobeJ (62). At least 200 cells were counted for each biological replicate. Notably and in agreement with prior studies, the presence of *prelA* reduced *E. coli* cell size by ~27% relative to *prelA’* in the absence of IPTG, suggesting that basal *relA* expression from an uninduced promoter is sufficient to promote division (Fig. 1A through D) (43, 44). *prelA’* behaved similarly to an empty vector control (*prelA-EV*) (Table S1; Fig. S1A), validating our use of *prelA’* as a negative control. We found that the difference in cell length mediated by *prelA* requires the presence of ppGpp’s RNAP binding partner, DksA. *prelA* resulted in only a ~15% reduction in the length of *dksA::Kan* mutant cells, a difference not statistically significantly different from the *dksA::Kan prelA’* control (*P* = 0.056) (Fig. 1E and F). However, we also noticed that the *dksA::Kan prelA’* cells were slightly longer than their wild-type (WT) counterparts (discussed in detail below), making it difficult to directly compare the effect of *prelA* between these genetic backgrounds. Importantly, the presence of *prelA* had no impact on cell width, consistent with a primary impact on division (Fig. S1B). The growth rates of *prelA* and *prelA’* strains were identical in the absence of an inducer (Fig. S1C and D), indicating that under these conditions, ppGpp mediates the length of wild-type cells independently of growth rate.

**Fig 2:**
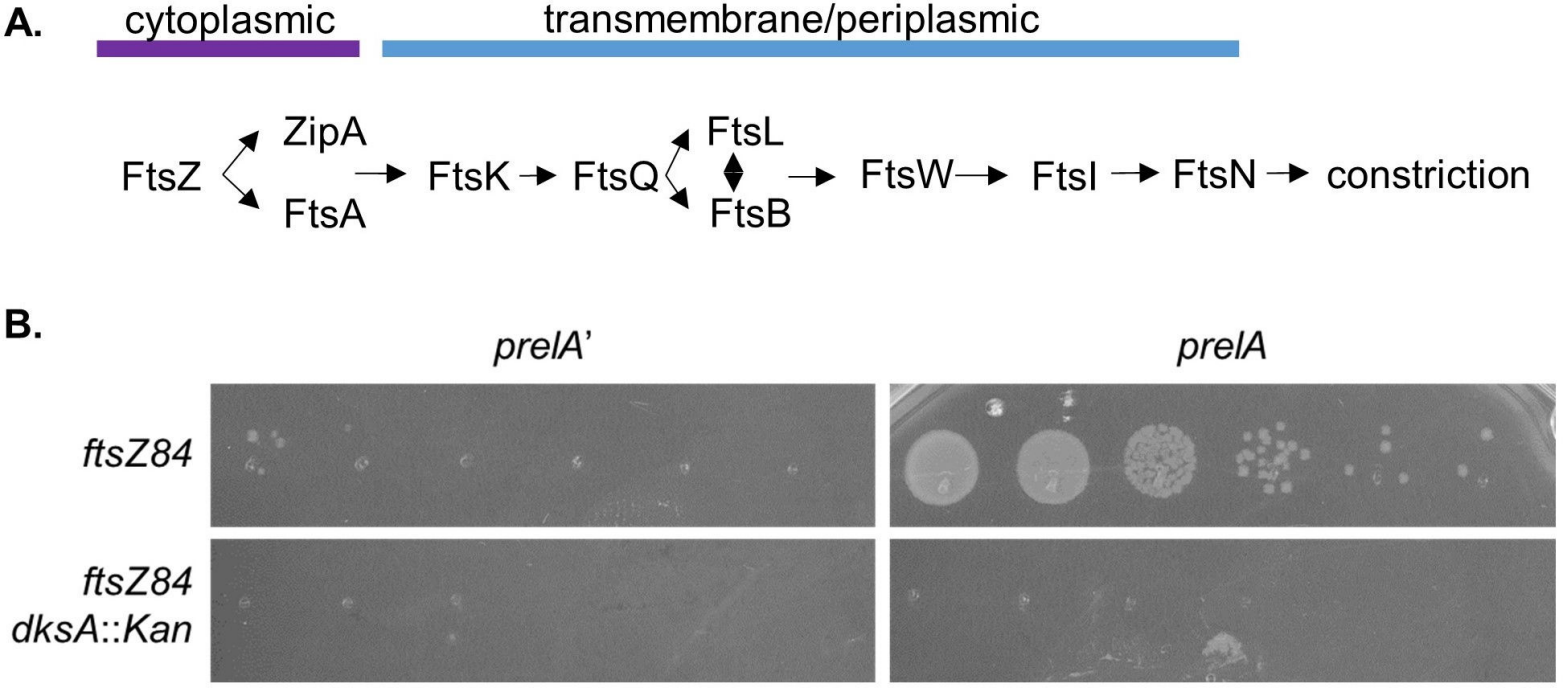
Excess ppGpp promotes division in a DksA-dependent manner. (A) Essential division proteins assemble hierarchically to midcell. (B) Expression of *prelA* facilitates growth of *ftsZ84*, but not *ftsZ84 dksA::Kan*, at 37°C. A representative image of three biological replicates is shown (no colonies are present for either *ftsZ84 dksA::Kan* strain).

To illuminate the impact of ppGpp on the activity of cell division proteins, we assessed the impact of excess ppGpp on the heat-sensitive alleles of five division genes: *ftsZ84* (G105S), *ftsA12* (A188V), *ftsQ1* (E125K), *ftsK44* (G80A), and *ftsI23* (Y380D) (35, 63–66). All five encode essential divisome components that assemble hierarchically at midcell (Fig. 2A) (28). While suppression of *ftsZ84* lethality by ppGpp has been reported (42), the effect of ppGpp on other conditional division mutants has not been examined. For these experiments, *prelA* was expressed without induction to slightly increase ppGpp levels, as above. To assess whether DksA is required for ppGpp’s documented effects on *ftsZ84* (42), we also expressed *prelA* in an *ftsZ84 dksA::Kan* double mutant. Strains were serially diluted and plated on LBNS without IPTG and incubated at 30^o^, 37^o^, and 42°C for 20 h prior to imaging.

Consistent with a role in ppGpp-mediated divisome activation, DksA was required for *prelA*-mediated suppression of *ftsZ84*. As expected based on a previous work (42), *prelA* increased CFUs of *ftsZ84* by approximately four orders of magnitude at the restrictive temperature of 37°C (Fig. 2B). In contrast, an *ftsZ84 dksA::Kan prelA* strain was unable to form colonies at 37°C (Fig. 2B). In addition, *prelA* did not result in consistent changes in the heat sensitivity of other conditional division alleles (Fig. S2), suggesting that ppGpp and DksA influence division specifically via FtsZ.

### ppGpp facilitates divisome assembly and/or activation during steady-state growth

**Fig 3:**
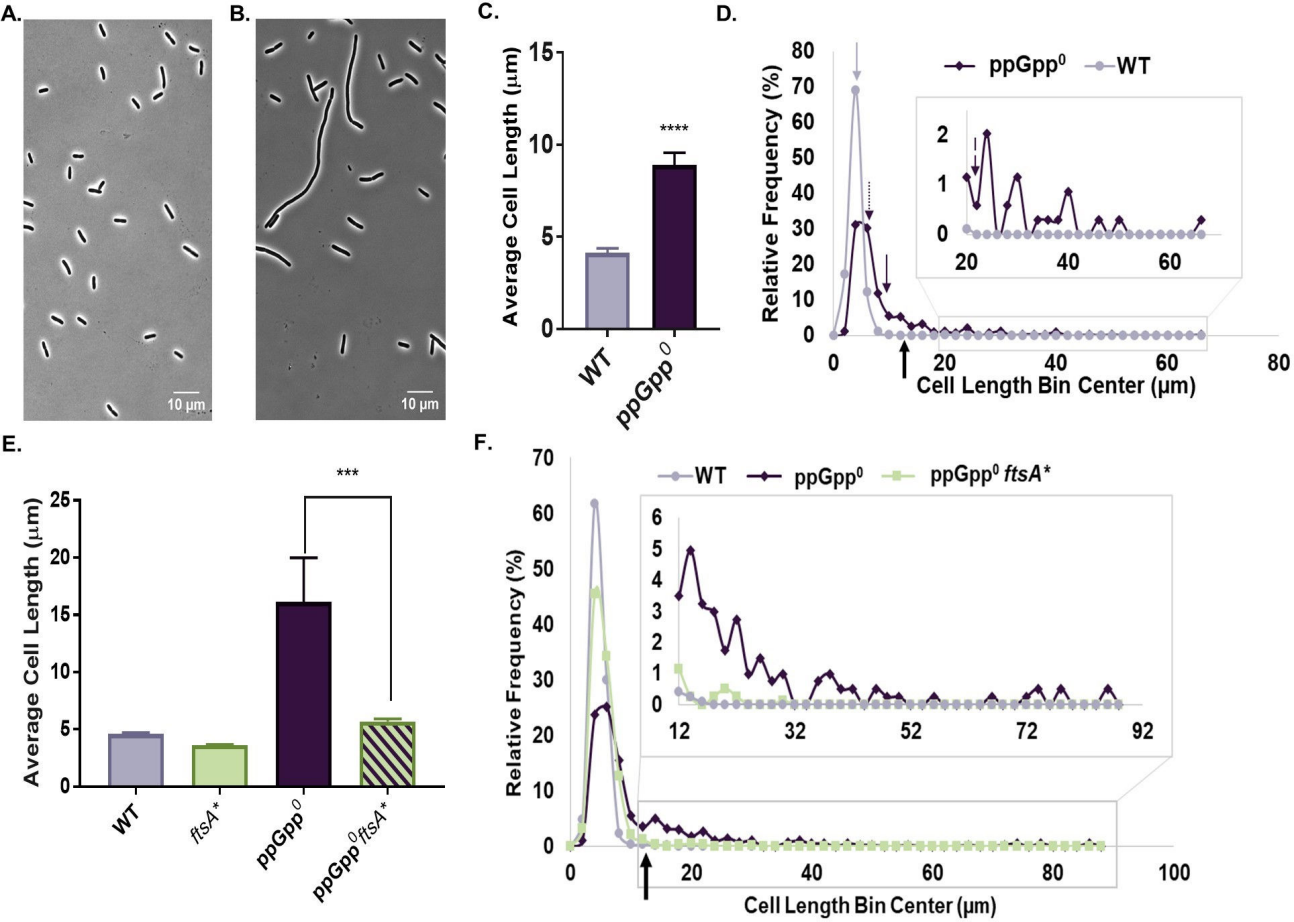
Basal ppGpp promotes cell division. (A, B) Representative phase contrast micrographs of WT (**A**) and Δ*relA spoT::cat* (ppGpp^0^) (**B**) cells. (C) ppGpp^0^ cells exhibit increased average cell length. Data represent averages and SDs of four biological replicates (****P ≤ 0.0001, two-tailed *t*-test). (D) Frequency distribution of individual cell lengths reveals both lengthening and filamentation among ppGpp^0^ cells. *N* > 200 cells from a representative biological replicate (bin width = 2 µm). The thick black arrow indicates the approximate location of the cut-off for filamentous cells (12.4 µm, 3× average wild-type length from Fig. 3C). The approximate location of the wild-type average (4.13 µm) is denoted by the light-purple arrow. The dark-purple arrows indicate the approximate location of the non-filamentous ppGpp^0^ average (6.02 µm, dotted line), the overall ppGpp^0^ average (8.89 µm, solid line), and the filamentous ppGpp^0^ average (22.06 µm, dashed line). (E) Average cell length of ppGpp^0^ ftsA* cells is significantly reduced compared with that of the ppGpp^0^ parent strain. Data represent averages and SDs of at least three independent replicates (***P ≤ 0.001 by one-way ANOVA with Tukey’s post-test). (F) Frequency distribution shows decreased lengthening and filamentation in ppGpp^0^ ftsA* cells. *N* > 400 cells from a representative replicate (bin width = 2 µm). The black arrow indicates the approximate location of the cut-off for filamentous cells (13.4 µm, 3× average wild-type length from Fig. 3E).

To clarify the contribution of ppGpp to division in nutrient replete conditions, we measured lengths of cells completely defective in ppGpp synthesis (Δ*relA spoT::cat*, hereafter ppGpp^0^) during growth in LB-glucose. As previously reported (19, 26), ppGpp^0^ mutants are heterogeneous for cell length; most ppGpp^0^ cells appeared slightly elongated compared with wild-type cells, while a sub-population of cells was highly filamentous (Fig. 3A and B). Considering the whole population, ppGpp^0^ cells were on average twofold longer than the wild-type parent (Fig. 3C).

To more fully characterize the impact of defects in ppGpp synthesis on cell length, we divided ppGpp^0^ cells into two populations using a cut-off of three times the average wild-type length to distinguish filaments from non-filaments (Fig. 3D). The non-filamentous subpopulation comprised ~80% of the ppGpp^0^ cells (Fig. 3D). Notably, non-filaments were 46% longer than the wild type (average cell length ~6.02 µm compared with 4.13 µm for the wild type), indicative of a significant defect in divisome assembly despite their relatively normal appearance. Filaments comprised ~20% of the ppGpp^0^ population (Fig. 3D). The filamentous population had an average length of 22.06 µm, over five times longer than the wild-type average. Filamentous cells were exceedingly rare in the wild-type control, with cells exceeding 20 µm not observed (Fig. 3D). ppGpp^0^ cells had similar widths to the wild type under the conditions tested, reinforcing a primary defect in division (Fig. S3A). The ppGpp^0^ mutant grew 19% slower than the wild-type in our conditions (Fig. S3B).

To confirm that lengthening and filamentation of ppGpp^0^ are a consequence of reductions in divisome assembly and/or activity, we took advantage of a gain-of-function mutation in the cell division protein FtsA, FtsA*. FtsA* can compensate for a variety of defects in divisome assembly, including the heat-sensitive alleles *ftsQ1* and *ftsK44*, as well as complete loss of the normally essential proteins ZipA, FtsK, or FtsN (39–41, 67). FtsA* also exhibits increased interactions with FtsZ and increased recruitment of FtsN compared with wild-type FtsA (39, 68, 69). If the increased length of ppGpp^0^ cells is due to destabilization of the core divisome, then we would expect ftsA* to restore divisome function and decrease cell length and filamentation in this background.

Supporting a role for basal ppGpp in promoting divisome assembly at a steady state, ftsA* reduced the average length of ppGpp^0^ (*relA::Kan spoT::cat*) cells ~65% compared with the ppGpp^0^ parent strain (Fig. 3E). ftsA* also largely eliminated the heterogeneity of ppGpp^0^ mutants, reducing the fraction of filamentous cells from 33% to 2% (Fig. 3F). ftsA* also reduced the average length of non-filamentous ppGpp^0^ cells by 21%, from 6.77 µm to 5.37 µm. ftsA* did not significantly change the ppGpp^0^ growth rate (Fig. S3C).

**Fig 4:**
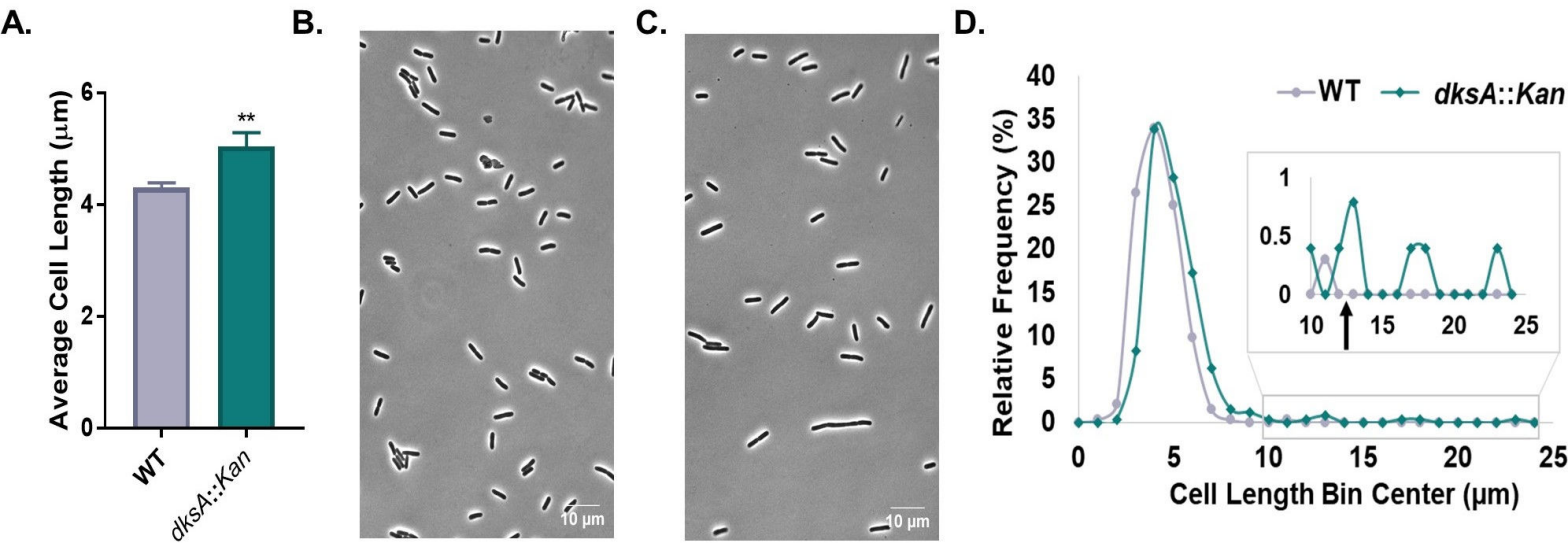
Deletion of *dksA* alone leads to cell lengthening. (A) *dksA::Kan* cells exhibit increased average cell length relative to the wild type. Data represent averages and SDs of four biological replicates (**P ≤ 0.01, two-tailed *t*-test). (B, C) Representative phase contrast micrographs of WT (**B**) and *dksA::Kan* (**C**) cells. (D) Frequency distribution of individual cells reveals lengthening and some filamentation by *dksA::Kan* cells compared with the wild type. *N* > 200 cells from a representative biological replicate (bin width = 1 µm). The black arrow indicates the approximate cut-off for designating cells as filamentous (12.9 µm, 3× wild-type cell length in Fig. 4A).

Finally, we also measured the effect of loss of *dksA* on the size in LB-glucose media. Defects in *dksA* had only a modest impact on cell length at a steady state in the presence of ppGpp, suggesting that the mere loss of the ppGpp-DksA-RNAP interaction is insufficient to explain the severe division defect in ppGpp^0^ cells. Non-filaments comprised ~99% of the *dksA::Kan* population (Fig. 4A through D), as compared with ~80% of a ppGpp^0^ population (Fig. 3D). On average, *dksA::Kan* mutant cells were 17% longer than the wild type (Fig. 4A). Non-filamentous *dksA::Kan* cells had an average length of 4.97 µm, a 15% increase over wild-type, but 17% shorter than the non-filamentous ppGpp^0^ cells measured in Fig. 3D. *dksA::Kan* filaments (~1% of the population) had an average length of 16.91 µm, a nearly fourfold increase compared with the wild type. *dksA::Kan* grew similarly to the wild type under the conditions tested (Fig. S4).

### ppGpp and DksA are required for optimal growth of conditional cell division mutants

**Fig 5:**
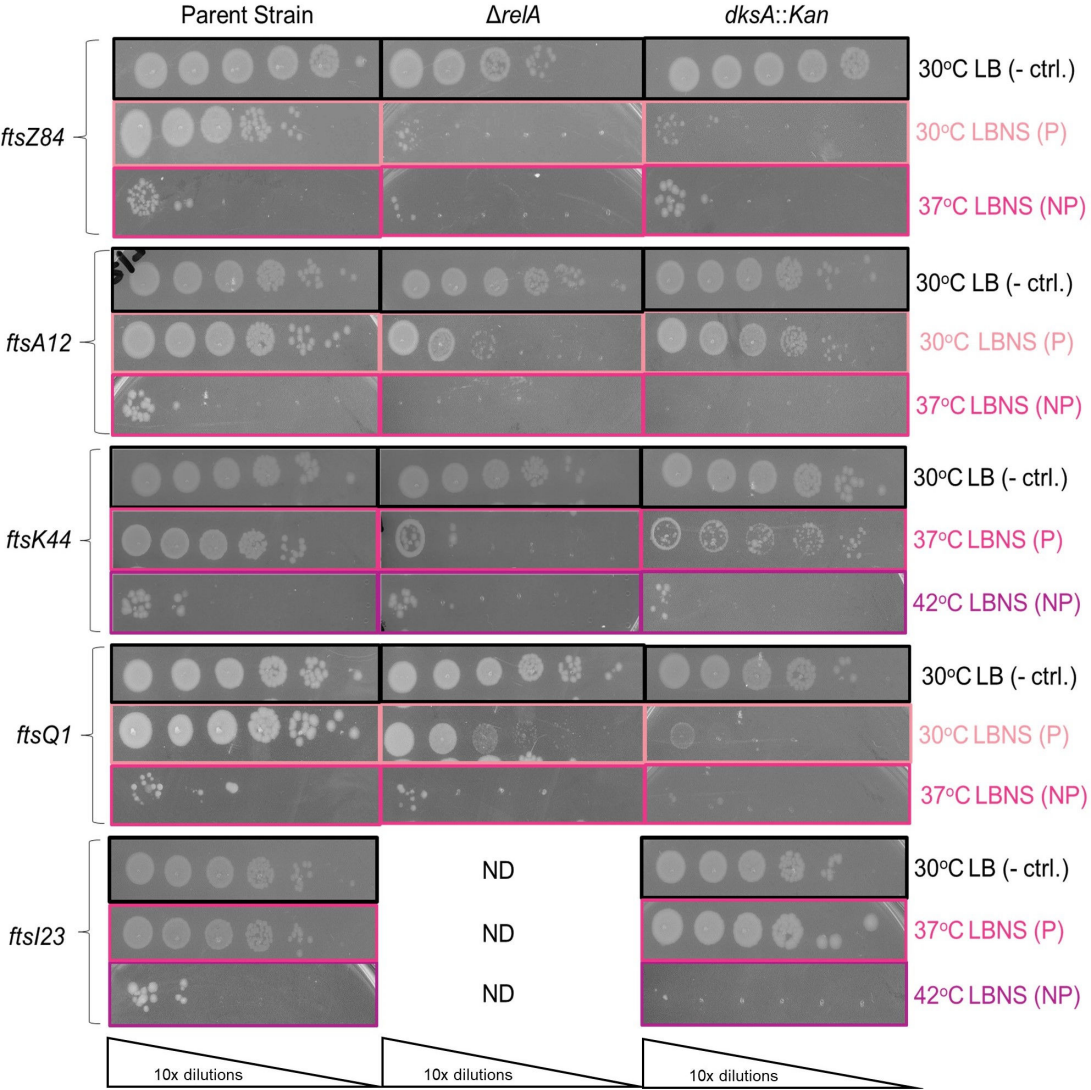
DksA and basal ppGpp are required for growth of conditional division mutants under permissive and non-permissive conditions. Effect of deletion of *relA* and *dksA* on heat-sensitive division mutants. Strains were grown on LBNS at temperatures that are permissive (P) or non-permissive (NP) for growth of the parent strain. Strains were also grown at 30°C on LB (- ctrl.) to confirm that an appropriate inoculum was used. Data shown are representative images of three biological replicates (ND, not determinable).

Further supporting a role for basal ppGpp as well as DksA in divisome activation, we found *relA* and *dksA* to be required for optimal growth of multiple heat-sensitive mutants under conditions permissive for growth of the parent strain (we were unable to obtain stable cell lines when we tried to combine heat-sensitive division alleles with mutations in both *relA* and *spoT*). Deletion of either *relA* (Δ*relA*, markerless deletion) or *dksA* (*dksA::Kan*) enhanced the heat sensitivity of *ftsZ84*, reducing CFUs at the permissive temperature of 30°C by roughly four orders of magnitude on LBNS (Fig. 5). Δ*relA* also reduced CFUs of *ftsK44* at the permissive temperature of 37°C by about three orders of magnitude and decreased the survival rate of *ftsA12* at both the permissive temperature of 30°C (by about two logs) and the non-permissive temperature of 37°C (no growth) (Fig. 5). Deletion of *relA* (*relA::Kan*) did not enhance heat killing of *ftsQ1* at the permissive temperature of 30°C; however, the colony size was reduced (Fig. 5). *dksA::Kan* eliminated the growth of both *ftsA12* and *ftsI23* at their non-permissive temperatures of 37°C and 42°C, respectively, and resulted in a roughly one log decrease in growth of *ftsK44* at the non-permissive temperature of 42°C (Fig. 5). *dksA::Kan* also decreased the colony size and reduced CFUs of *ftsQ1* by about one order of magnitude at the permissive temperature of 30°C (Fig. 5). The effect of *relA::Kan* on *ftsI23* could not be determined due to the rapid accumulation of suppressors in the double mutant.

### DksA requires ppGpp to promote division

**Fig 6:**
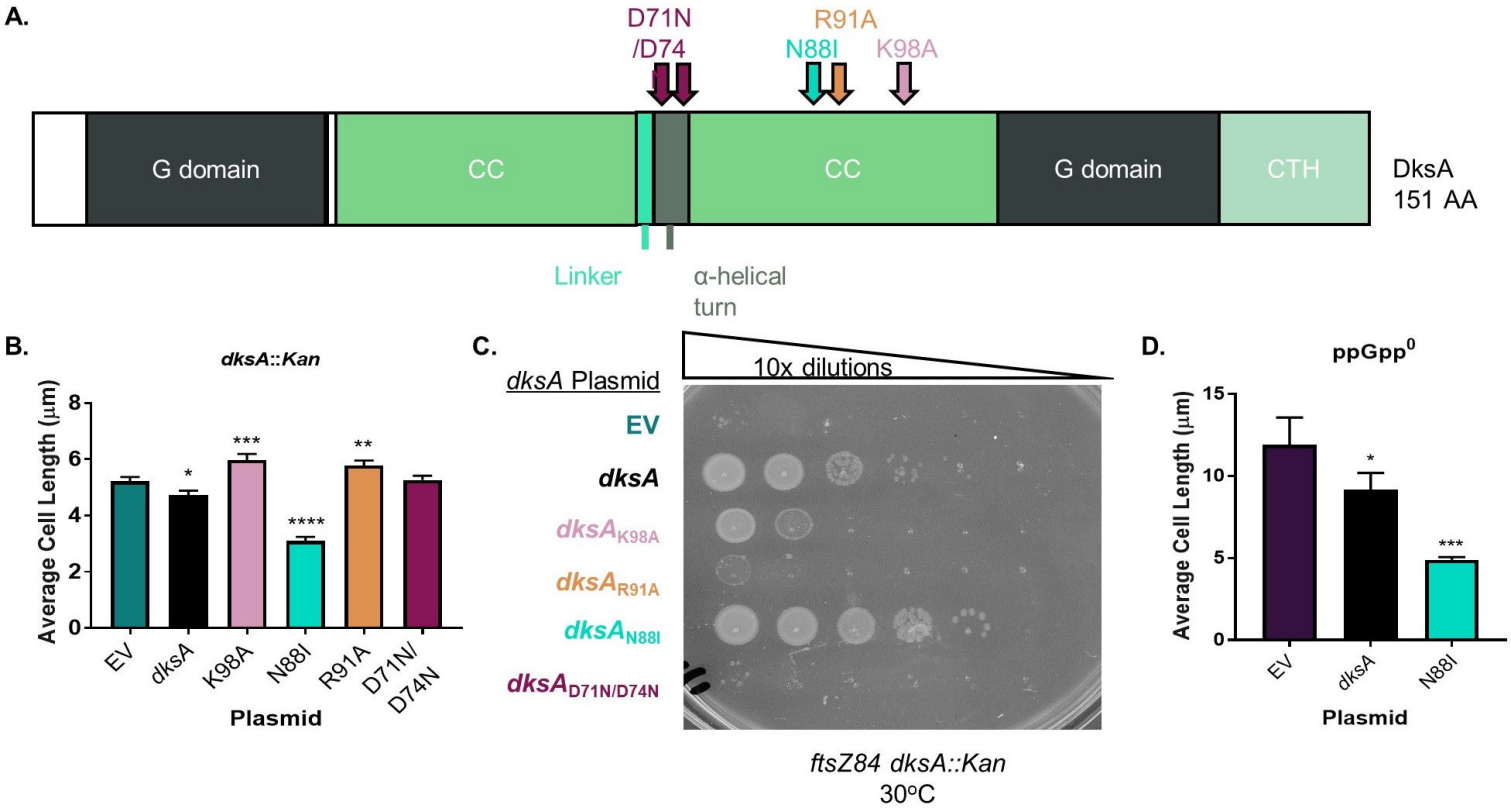
Mutant analysis indicates that DksA requires ppGpp and RNAP binding to promote division. (A) Locations of separation-of-function mutations in DksA. DksA is comprised of a globular (G) domain, a coiled coil domain (CC) connected by a linker and α-helical turn, and a C-terminal helix (CTH) (70). In the α-helical turn, D71N/D74N interferes with transcriptional regulation by DksA. In the CC domain, N88I enhances DksA’s affinity for RNAP, R91A reduces DksA binding to RNAP, and K98A blocks binding of ppGpp to DksA. (B) Complementation of cell length in a *dksA::Kan* mutant by different alleles of *dksA* expressed from a plasmid reveals that cell length control by DksA requires binding to ppGpp and RNAP. Data represent averages and SDs from three biological replicates (*P ≤ 0.05, **P ≤ 0.01, ***P ≤ 0.001, and ****P ≤ 0.0001 relative to empty vector control by one-way ANOVA with Dunnett’s post-test). (C) Complementation of enhanced *ftsZ84* heat sensitivity of a *dksA::Kan* mutant by different *dksA* alleles expressed from a plasmid supports a role for ppGpp and RNAP binding by DksA in the activation of FtsZ. A representative image of three biological replicates is shown. (D) Effect of *dksA* alleles expressed from a plasmid on length of ppGpp^0^ cells shows that a gain-of-function *dksA* mutant can complement lengthening of ppGpp^0^ cells better than wild-type *dksA*. Data represent averages and SDs from three biological replicates (*P ≤ 0.05 and ***P ≤ 0.001 relative to EV by one-way ANOVA with Dunnett’s post-test).

As noted in the introduction, interaction with ppGpp alters the DksA-RNAP interaction, and DksA displays different effects on transcription in the presence and absence of ppGpp (14, 16). To further illuminate the impact of ppGpp on DksA-mediated division effects, we took advantage of plasmids expressing separation-of-function *dksA* alleles affected for ppGpp binding (gift of R. Gourse) (Table 1; Table S1; Fig. 6A). These alleles include *dksA*_K98A_, which blocks binding of ppGpp to RNAP Site 2, and *dksA*_N88I_, a “super” DksA allele with increased affinity for RNAP that can rescue some phenotypes associated with loss of ppGpp (8, 54).

Analysis of cell length and *ftsZ84* heat sensitivity support a model in which ppGpp binding promotes DksA-dependent division activation. As expected, complementation of *dksA::Kan* with wild-type *dksA* significantly decreased length by 9% (Fig. 6B; Fig. S5A). The same plasmid also restored growth of *ftsZ84 dksA::Kan* at 30°C, increasing CFUs by three orders of magnitude (Fig. 6C). In contrast, a plasmid encoding the ppGpp “blind” *dksA* allele, *pdksA*_K98A_, failed to reduce the length of *dksA::Kan* cells (Fig. 6B; Fig. S5A) and resulted in only partial complementation of *ftsZ84 dksA::Kan*; CFUs were increased by about three logs, but colonies were extremely small at 30°C (Fig. 6C). Conversely, the super DksA, *dksA*_N88I_, which can compensate for some ppGpp^0^ phenotypes (54), decreased the *dksA::Kan* cell length by ~40%, even more than the wild type (Fig. 6B; Fig. S5A). This mutant also increased CFUs of *ftsZ84 dksA::Kan* by four orders of magnitude at 30°C (Fig. 6C). The growth rate of *dksA::Kan* mutants was unaffected by complementation with either the wild-type or mutant *dksA* alleles (Fig. S5B).

As a final confirmation that ppGpp activates division through DksA, we expressed the super DksA, *dksA*_N88I_, in ppGpp^0^ cells and measured the impact on length. *pdksA*_N88I_ reduced the cell length of ppGpp^0^ by 58%, while *pdksA* reduced the length by only 23% (Fig. 6D). *pdksA*_N88I_ reduced the percentage of filaments from 27% to ~1%, while *pdksA* only reduced filamentation to 17% (Fig. S5C). The finding that a gain-of-function DksA allele strongly suppresses the division defect of ppGpp^0^ cells suggests that the positive impact of ppGpp on division is mediated through DksA. Both *pdksA* and *pdksA*_N88I_ increased the growth rate of ppGpp^0^ (Fig. S5D). Altogether these experiments strongly indicate that ppGpp binding is required for activation of division by DksA.

### DksA functions as a division inhibitor in the absence of ppGpp

**Fig 7:**
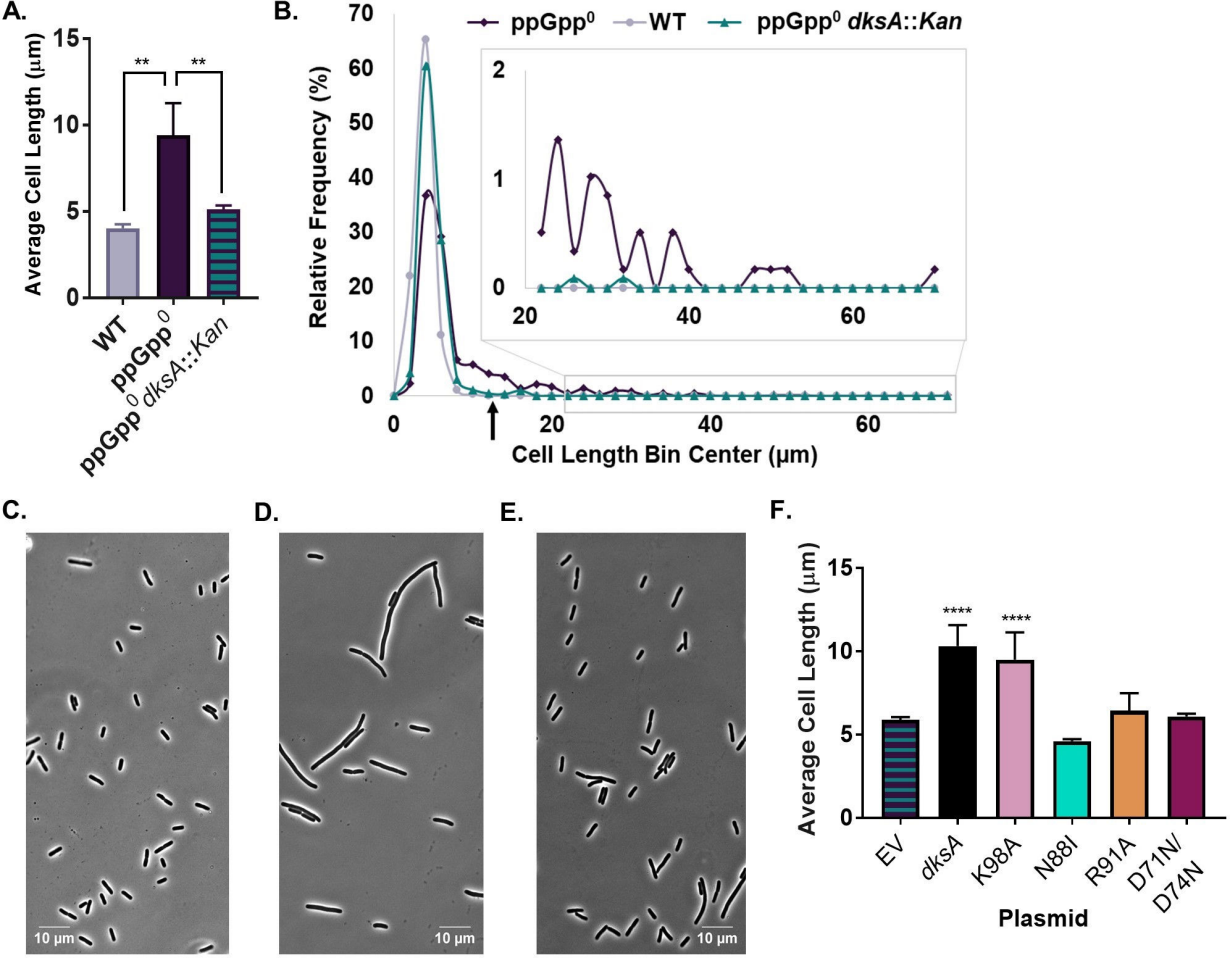
In the absence of ppGpp, DksA behaves as a division inhibitor. (A) Deletion of *dksA* in the ppGpp^0^ mutant reduces the average cell length. Data represent averages and SDs of three independent replicates (**P ≤ 0.01 relative to ppGpp^0^ by one-way ANOVA with Dunnett’s post-test). (B) Frequency distribution of single cell lengths demonstrates that ppGpp^0^ *dksA::Kan* exhibits less lengthening and filamentation than ppGpp^0^, although cells are still longer and exhibit more filaments than the wild type. *N* > 500 cells from a representative experiment (bin width = 2 µm). The black arrow indicates the approximate location of the cut-off for filamentous cells (12.2 µm, 3× wild-type length in Fig. 7A). (C–E) Representative phase contrast micrographs of WT (**C**), ppGpp^0^ (**D**), and ppGpp^0^ *dksA::Kan* (**E**) cells. (F) Complementation of decreased length in ppGpp^0^ *dksA::Kan* mutants by different alleles of *dksA* expressed from plasmids reveals a requirement for RNAP binding by DksA. Data represent averages and SDs of at least three biological replicates (****P ≤ 0.0001 relative to empty vector control by one-way ANOVA with Dunnett’s post-test).

To gain a full picture of the effect of DksA and ppGpp on division, we measured the length of ppGpp^0^ *dksA::Kan* triple-mutant cells. To our surprise, our results suggested that DksA inhibits division in the absence of ppGpp. ppGpp^0^ *dksA::Kan* exhibited a 1.8-fold decrease in average cell length compared with ppGpp^0^ (Fig. 7A). This decrease in length was driven both by shortening of non-filamentous cells and by a decrease in the number of filaments. Non-filamentous ppGpp^0^ *dksA::Kan* cells had an average length of 4.81 µm, compared with 5.67 µm for non-filamentous ppGpp^0^ and 4.03 µm for the wild type. The frequency of filaments was reduced from 21% in ppGpp^0^ to 3% in ppGpp^0^ *dksA::Kan* (Fig. 7B through E). Altogether, ppGpp^0^ *dksA::Kan* looked more similar to *dksA::Kan* than ppGpp^0^, suggesting that DksA may be epistatic to ppGpp in terms of cell length (Fig. 4). The ppGpp^0^ *dksA::Kan* mutant also exhibited a faster growth rate than the ppGpp^0^ parent (Fig. S6).

To further confirm that DksA negatively impacts division when not bound to ppGpp, we complemented ppGpp^0^ *dksA::Kan* with plasmids encoding wild-type *dksA*, the ppGpp blind allele, *dksA*_K98A_, and the super DksA, *dksA*_N88I_, and measured effects on cell length. As expected, a plasmid encoding wild-type *dksA* increased the cell length of ppGpp^0^ *dksA::Kan* 1.8-fold and increased the frequency of filamentation from 3% to 18% (Fig. 7F; Fig. S7A). Complementation with the wild-type allele also reduced the growth rate of ppGpp^0^ *dksA::Kan* (Fig. S7B). Complementation of ppGpp^0^ *dksA::Kan* with the ppGpp blind allele, *pdksA*_K98A_*,* also increased the cell length by 1.6-fold and increased the frequency of filaments to 16% (Fig. 7F; Fig. S7B). In contrast, the super DksA, *pdksA*_N88I_, failed to complement cell length and actually reduced filamentation to ~0.5% (Fig. 7F; Fig. S7B). These data support a model in which ppGpp binding switches DksA from negatively to positively influencing division.

### DksA and ppGpp indirectly regulate the divisome through transcription

Although substantial transcriptomics data argue against either ppGpp or DksA as direct regulators of division gene expression, the most straightforward explanation of their positive influence on division is via interaction with RNAP. To probe this idea, we complemented *dksA::Kan*, *dksA::Kan ftsZ84*, and ppGpp^0^ *dksA::Kan* mutants with plasmids encoding *dksA* alleles defective for interactions with RNAP (Table 1; Table S1). These alleles include the following: *dksA*_R91A_, which has reduced affinity for RNAP, and *dksA*_D71N/D74N_, which still binds RNAP but is defective for regulating transcriptional initiation by RNAP (8, 55, 56) (Fig. 6A).

Altogether our results support a model in which DksA and ppGpp regulate division through interaction with RNAP and downstream effects on transcription. In contrast to wild-type *dksA*, both *dksA*_R91A_ and *dksA*_D71N/D74N_ failed to complement the cell length associated with *dksA::Kan* (Fig. 6B; Fig. S5A) or facilitate growth of *ftsZ84 dksA::Kan* at 30°C (Fig. 6C). *pdksA*_R91A_ and *pdksA*_D71N/D74N_ also failed to increase length or the rate of filamentation of ppGpp^0^ *dksA::Kan* (Fig. 7F; Fig. S7B).

### A ppGpp binding defective RNAP_1−2−_ mutant does not phenocopy length and division phenotypes associated with loss of ppGpp

To clarify whether ppGpp activates division through transcription, we measured cell length and *ftsZ84* heat sensitivity in a strain containing mutations in *rpoZ* and *rpoC* that block binding of ppGpp to RNAP (designated RNAP_1−2−_) (8). RNAP_1−2−_ mutant failed to phenocopy a ppGpp^0^ strain, exhibiting no change in length or filamentation compared with an isogenic wild-type control (RNAP_1+2+_) (Fig. S8A and B). A RNAP_1−2−_ *ftsZ84* mutant exhibited a roughly one log decrease in CFUs at the permissive temperature of 30°C (Fig. S8C), a much milder change than that caused by Δ*relA* (Fig. 5). Furthermore, the RNAP_1−2−_ *ftsZ84* strain actually exhibited a two log increase in CFU at the non-permissive temperature of 37°C, again in contrast to *ftsZ84* Δ*relA* (Fig. S8C and D). The RNAP_1−2−_ mutant does not affect binding of ppGpp to other cellular targets, so this could suggest that other ppGpp targets may also be involved in division. However, our other data clearly indicate that ppGpp regulates division in a large part through its effects on DksA and RNAP. Available transcriptomics data do not provide an obvious explanation for the phenotypic discrepancy between RNAP_1−2−_ and ppGpp^0^ (11).

### ppGpp and DksA promote recruitment of divisome proteins to the nascent septum

Altogether, our data suggest ppGpp and DksA work coordinately to modulate cell division during both exponential growth and nutrient stress. To illuminate the step at which they function, we measured recruitment of fluorescently labeled division proteins to the nascent septum in wild-type, ppGpp^0^, *dksA::Kan*, and ppGpp^0^ *dksA::Kan* strains. In wild-type cells, the divisome complex forms a ring-like structure at midcell, which can be visualized as a bright band of fluorescence using GFP-labeled proteins (Fig. S9A through E).

For these experiments, we expressed *gfp-*tagged, IPTG-inducible alleles of the essential cell division genes *ftsZ*, *ftsA*, *ftsL*, *ftsI*, and *ftsN* from the lambda locus in strains also encoding a wild-type copy of that gene, as described previously (33). Strains were grown in LB IPTG and fixed prior to imaging. To quantify changes in division protein recruitment between strains, we calculated the “length-to-ring ratio” (*L*/*R*), which is the total cell length for the entire population divided by the total number of rings. An increase in *L*/*R* indicates a defect in division protein recruitment.

**Fig 8:**
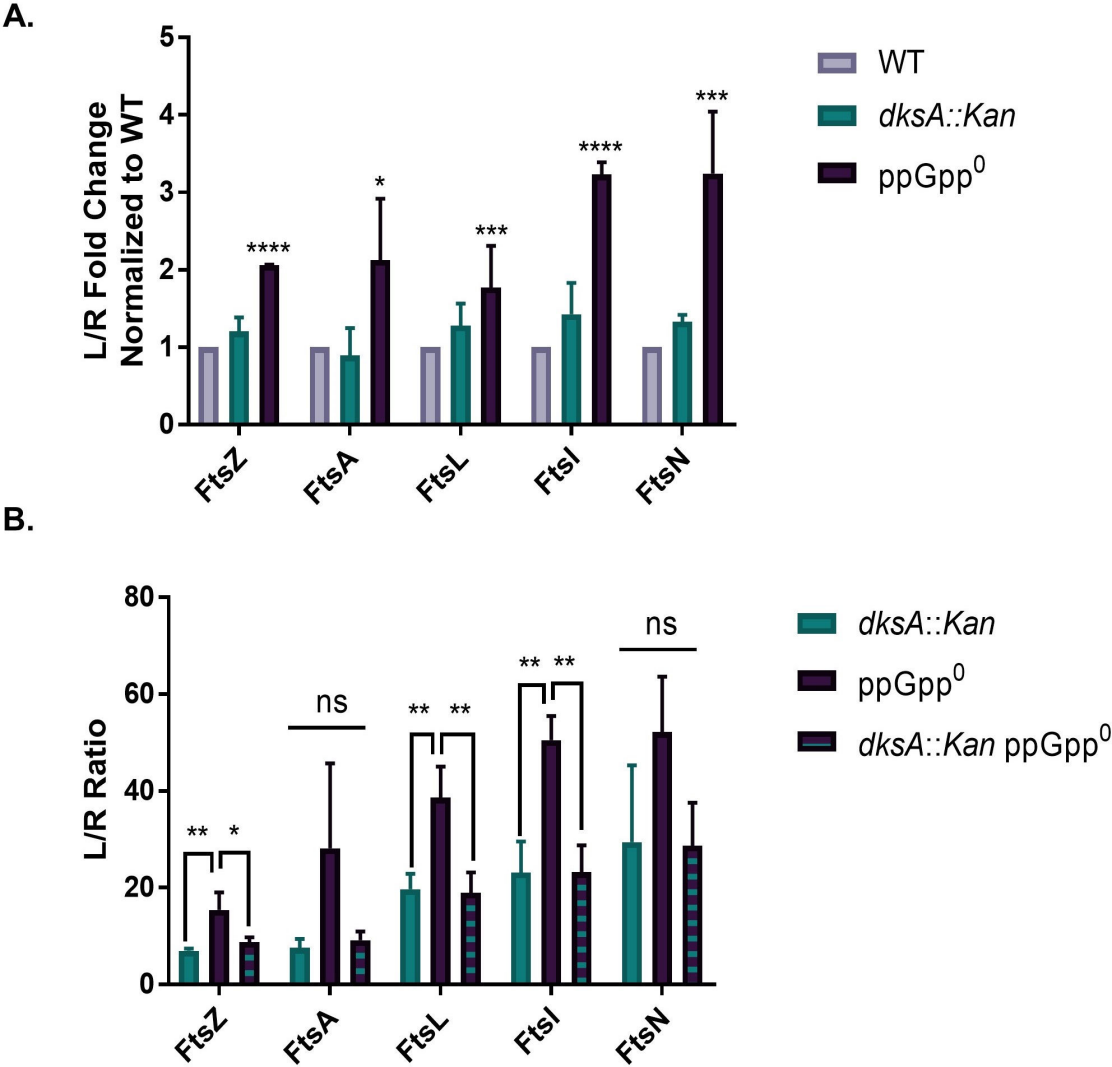
ppGpp and DksA modulate division protein recruitment. (A) Fold changes of length/ring ratios for GFP-tagged division proteins expressed in *dksA::Kan* and ppGpp^0^ cells relative to the wild type. Data represent averages and SDs of three independent replicates (*P ≤ 0.05, ***P ≤ 0.001, and ****P ≤ 0.0001 relative to WT by one-way ANOVA of untransformed data with Dunnett’s post-test). (B) Length/ring ratios of GFP-tagged division proteins expressed in *dksA::Kan*, ppGpp^0^, and ppGpp^0^ *dksA::Kan* cells. Data represent averages and SDs of three biological replicates (ns, not significant; *P ≤ 0.05 and **P ≤ 0.01 by one-way ANOVA with Tukey’s multiple comparisons test).

Supporting DksA as a division inhibitor in the absence of ppGpp, ppGpp^0^ cells exhibit DksA-dependent defects in divisome recruitment. ppGpp^0^ exhibited significant two- to threefold increases in *L*/*R* for all division proteins tested, indicating defects in divisome assembly (Fig. 8A). Filamentous ppGpp^0^ cells usually had only one or two rings of each division protein, and those rings were typically located near the ends of filaments (Fig. S9). *dksA::Kan* exhibited no changes in *L*/*R* for any division proteins tested compared with the wild type (Fig. 8A). On the other hand, ppGpp^0^ *dksA::Kan* exhibited significant ~2-fold reductions in *L*/*R* of GFP-FtsZ, GFP-FtsL, and GFP-FtsI compared with ppGpp^0^ (Fig. 8B). Similar trends were observed for GFP-FtsA and GFP-FtsN, although differences were not statistically significant. For all division proteins, *L*/*R* was indistinguishable between *dksA::Kan* and ppGpp^0^ *dksA::Kan*, indicating that DksA is epistatic to ppGpp in terms of division protein recruitment. These results suggest that in the absence of ppGpp, DksA inhibits divisome assembly, but ppGpp activates assembly by binding DksA and relieving this inhibition.

### DksA and ppGpp likely modulate divisome assembly through their impact on recruitment of FtsZ

Thus far, our data support a model in which transcriptional changes mediated by ppGpp and DksA lead to indirect activation (DksA + ppGpp) or inhibition (DksA alone) of divisome assembly. At the same time, the hierarchical nature of divisome assembly makes it difficult to know which proteins are the primary target. To clarify this issue, we examined the effect of overexpressing a collection of division genes on cell length in *dksA::Kan* and ppGpp^0^ backgrounds.

**Fig 9:**
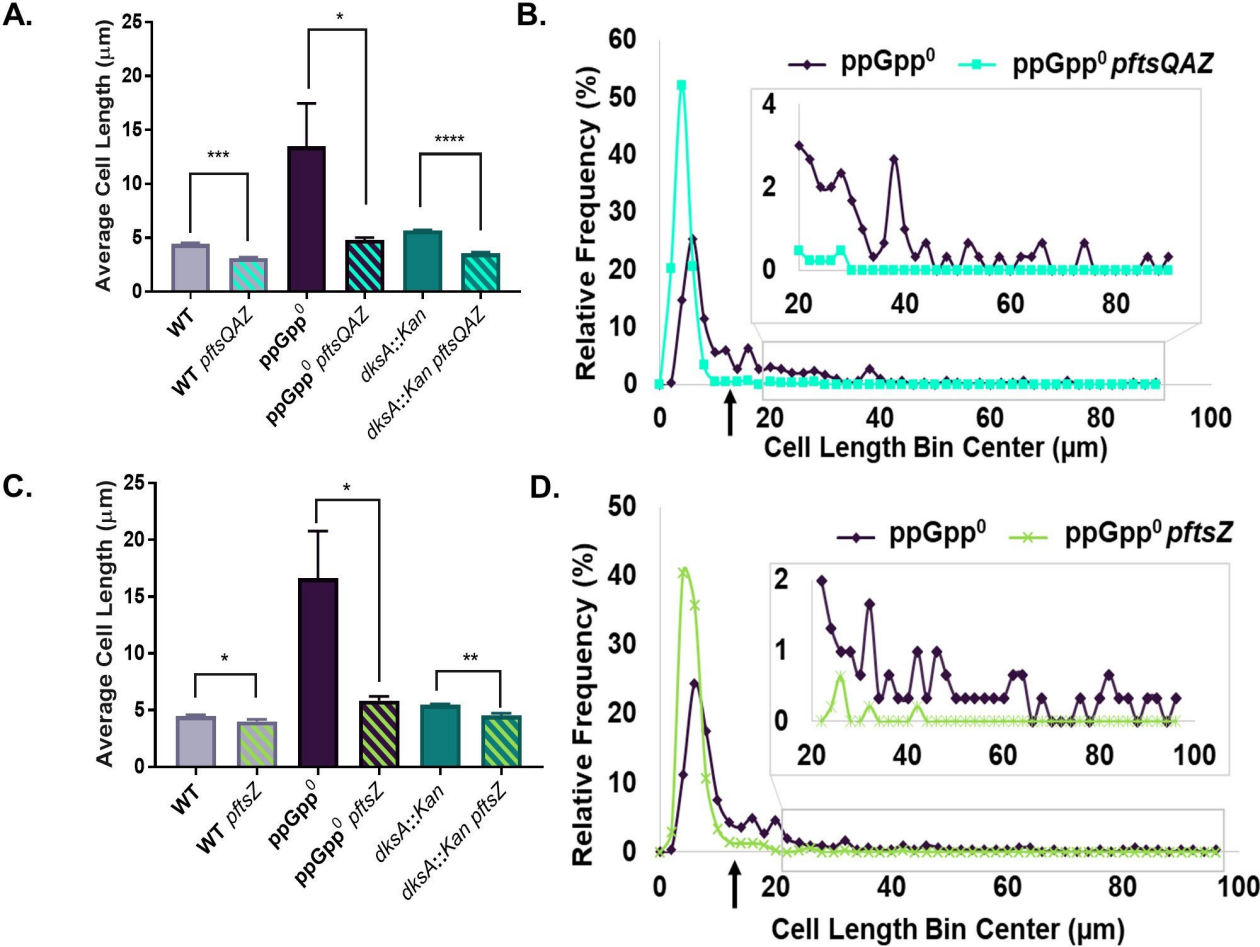
Ectopic expression of *ftsZ* reduces the length of ppGpp^0^ and *dksA::Kan*. (A) Overexpression of the *ftsQAZ* operon leads to decreased average cell length in ppGpp^0^ and *dksA::Kan* cells relative to vector-free controls. Data represent averages and SDs of three biological replicates (*P ≤ 0.05, ***P ≤ 0.001, and ****P ≤ 0.0001 by two-tailed *t*-test). (B) Frequency distribution of individual cell lengths shows that overexpression of *ftsQAZ* decreases both lengthening and filamentation among ppGpp^0^ cells. *N* > 200 cells from a single representative experiment (bin width = 2 µm). The black arrow indicates the approximate cut-off for filamentous cells (13.4 µm, 3× the wild-type average in Fig. 9A). (C) Overexpression of *ftsZ* alone reduces average wild-type, ppGpp^0^, and *dksA::Kan* cell length relative to vector-free controls. Data represent averages and SDs of three biological replicates (*P ≤ 0.05 and **P ≤ 0.01 by two-tailed *t*-test). (D) Frequency distribution of individual cell lengths shows that overexpression of *ftsZ* alone reduces lengthening and filamentation among ppGpp^0^ cells. *N* > 300 cells from a single representative experiment (bin width = 2 µm). The black arrow indicates the approximate cut-off for filamentous cells (13.5 µm, 3× the wild-type average in Fig. 9C).

Supporting FtsZ as a primary target, the presence of a plasmid encoding *ftsZ* under the control of its native promoter was sufficient to reduce the length of both *dksA::Kan* and ppGpp^0^ cells. We first used a plasmid expressing the *ftsQAZ* operon from its native promoter (59) (Table 1; Table S1), which resulted in a ~2-fold increase in FtsZ levels (Fig. S10). This plasmid reduced the length of ppGpp^0^ mutants by 2.8-fold and reduced the length of *dksA::Kan* cells by 37% (Fig. 9A). *pftsQAZ* also reduced the proportion of filaments in the ppGpp^0^ background from 30% to 3% (Fig. 9B). Similar results were obtained with a plasmid encoding only *ftsZ*, which reduced length of ppGpp^0^ and *dksA::Kan* by 2.8-fold and 17%, respectively, and reduced filamentation of ppGpp^0^ from 37% to 3% (Fig. 9C and D). We were unable to detect a statistically significant increase in FtsZ levels due to this plasmid, suggesting that very mild changes in FtsZ levels are sufficient to reduce lengthening and filamentation (Fig. S10). *pftsQAZ* and *pftsZ* also reduced the wild-type cell length by 30% and 11%, respectively (Fig. 9A and B). No changes in growth rate were observed for strains expressing *pftsQA* or *pftsZ* (Fig. S11). A plasmid expressing only *ftsQA* failed to significantly affect the size in any background, indicating these genes are unlikely to be directly involved in ppGpp-mediated division activation (Fig. S12A). Similarly, expression of *gfp-ftsN* from an IPTG-inducible promoter, which reduces the length of wild-type cells (33), had no impact on ppGpp^0^ cell length (Fig. S12B). This demonstrates that FtsN, the last protein recruited to the division site, is not sufficient to compensate for division defects in ppGpp^0^ cells.

### ppGpp and DksA modulate division independently of detectable effects on FtsZ concentration

Because different groups have reported different effects of ppGpp on FtsZ protein levels (27, 42, 45) and our results suggest that FtsZ is a major target of regulation by DksA and ppGpp, we wanted to clarify whether DksA and ppGpp regulate FtsZ levels via quantitative immunoblotting. We quantified FtsZ concentrations in wild-type, ppGpp^0^, *dksA::Kan*, and ppGpp^0^ *dksA::Kan*, as well as in wild-type cells expressing *prelA*, *prelA’*, or *pftsQAZ*. We found that DksA and ppGpp have no detectable effect on FtsZ levels. Only the *pftsQAZ*-positive control exhibited a significant change in FtsZ concentration compared with the wild type (Fig. S13). This suggests that although ppGpp and DksA influence divisome assembly through FtsZ, they do not appear to regulate FtsZ levels.

## DISCUSSION

**Fig 10:**
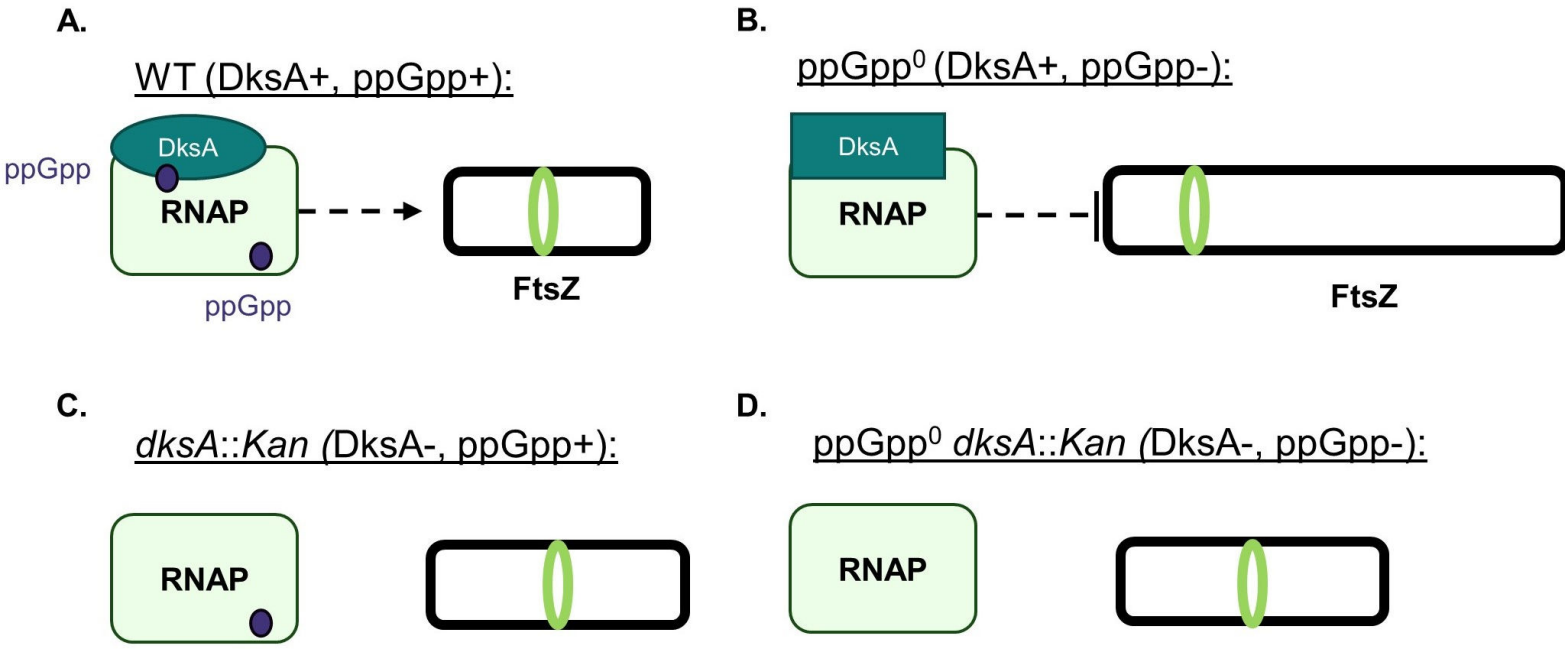
Model of division regulation by ppGpp and DksA. (A) In wild-type cells, ppGpp and DksA are both available to bind RNAP. The ppGpp/DksA/RNAP complex indirectly activates FtsZ, resulting in activation of division and maintenance of wild-type cell size. (B) In the absence of ppGpp, DksA switches to behaving as an indirect inhibitor of FtsZ, resulting in cell lengthening and filamentation. (C) In the absence of *dksA*, ppGpp is no longer able to activate division, resulting in lengthening of *dksA::Kan* cells compared with the wild type. (D) In the absence of both ppGpp and DksA, no indirect transcriptional regulation of division occurs. This results in decreased length compared with ppGpp^0^ and similar length to *dksA::Kan*. Cell length depictions are not to scale.

This study establishes ppGpp and DksA as critical modulators of cell length and division in *E. coli*. Most importantly, when bound to ppGpp, DksA interacts with RNAP to promote assembly of the division machinery and reduce cell length (Fig. 10A). Loss of *dksA* alone leads to increased cell length (Fig. 4 and 10C). While extensive transcriptomics data suggest ppGpp-DksA’s impact on division is indirect and independent of changes in division gene expression (11, 16, 45, 49, 50), genetic data suggest FtsZ is the eventual downstream target (Fig. 9). Unexpectedly, in the absence of ppGpp, DksA has an inhibitory effect on division (Fig. 10B). Deletion of *dksA* in the absence of ppGpp significantly reduces the fraction of filamentous cells in the population and also reduces the average length of the non-filamentous fraction (Fig. 7 and 10D). Genetic and cytological analyses suggest that DksA exerts an inhibitory effect on division via changes in recruitment and/or activation of FtsZ (Fig. 9). Transcriptomic data indicate that the *negative* impact of DksA on division in the absence of ppGpp is also likely indirect (16, 49).

Our results extend previous work suggesting a link between ppGpp and division. Specifically, we found that ppGpp exerts a positive impact on the assembly of the division machinery in a manner that requires interactions with both DksA and RNAP (Fig. 1, 2 and 6), indicating that ppGpp indirectly regulates division through its effects on transcription. We also established the importance of basal ppGpp levels in maintaining the wild-type cell size and modulating division. While previous work showed that cells lacking ppGpp sometimes filament (19, 26), this study established that filaments comprise approximately 20% of ppGpp^0^ populations grown in rich media (Fig. 7) (a value that may be slightly underestimated due to the higher probability of long filaments being excluded from cell length calculations due to extending past the field of view or being tangled with other cells). We further uncovered that non-filamentous ppGpp^0^ cells are still longer than the wild type, consistent with a division defect in these cells.

Although collectively our data indicate that ppGpp’s major effects on division are mediated through transcription (Fig. 1, 2, and 6), an RNAP_1−2−_ mutant unable to bind ppGpp does not recapitulate the phenotype of a ppGpp^0^ mutant with regard to cell length or hypersensitivity of *ftsZ84* (Fig. S8). The RNAP_1−2−_ mutant has a high number of point mutations in multiple polymerase genes: a three-codon deletion in *rpoZ* and five different codon substitutions in *rpoC* (all from charged or polar amino acids to alanine) (8, 11). It is possible that these mutations result in pleiotropic changes that mask the effects of loss of ppGpp binding on division. As this is not the only example of phenotypic differences between ppGpp^0^ and RNAP_1−2−_ strains (7, 8), discrepancies between these mutants should be explored further.

Our finding that DksA exhibits opposing effects on division depending on the presence of ppGpp was also unexpected, although there is some precedent for DksA and ppGpp exerting divergent functions in the cell. While ppGpp and DksA are typically thought to work cooperatively to influence transcription, loss of ppGpp or *dksA* leads to opposing transcriptional changes for a subset of genes (16, 49). DksA regulates polyphosphate accumulation in a ppGpp-independent manner (18). ppGpp^0^ and *dksA* mutants have also been reported to exhibit some divergent phenotypes; for example, loss of ppGpp decreases fimbriation and fimbriae-dependent adhesion, whereas loss of *dksA* increases these phenotypes (17, 19). However, to our knowledge, this report is the first example of DksA itself having opposite effects on a single phenotype depending on ppGpp.

The mechanism underlying these contrasting effects remains unclear. Binding of DksA to RNAP results in conformational changes in both, leading to mechanical stress (14). Binding of ppGpp to Site 2 induces further conformational changes that relieve this stress and position the coiled-coil domain of DksA closer to the RNAP active site (14). Together with previous work indicating that the ppGpp and DksA regulons do not completely overlap, our data suggest that these two conformations of DksA may have different transcriptional outputs, which lead to opposing effects on cell division. Alternatively, it is also possible that differences in the phenotypes between the ppGpp^0^ and ppGpp^0^ *dksA::Kan* strains are not due to direct transcriptional effects by DksA in the absence of ppGpp, but rather to increased binding of GreA and/or GreB in the absence of DksA. These proteins compete with DksA for the same binding site on RNAP, and their increased binding in the absence of DksA has transcriptional consequences (49). Further work is needed to discriminate between these possible mechanisms.

Our work suggests a mechanism in which the ratio of DksA bound to ppGpp versus DksA unbound from ppGpp modulates cell division and dictates cell length. ppGpp levels vary with the nutritional state, growth phase, and presence of stressors (3), while DksA levels remain constant (and in excess of RNAP levels) throughout growth (12, 57, 71). In the absence of nutritional stress, we propose that steady basal levels of ppGpp allow for the ratio of RNAP bound to DksA/ppGpp vs. DksA alone to be maintained at a certain level, leading to the maintenance of cell size. As ppGpp levels increase during stress conditions, more DksA should be in its ppGpp-bound, division-activating state, leading to a decreased cell length. This effect is likely enhanced by the fact that ppGpp increases DksA’s affinity for RNAP (14, 72). In cells with lower than normal ppGpp levels or cells lacking ppGpp altogether, the high level of DksA bound to RNAP alone should lead to division inhibition and increased length. Consistent with the observation that length is strongly negatively correlated with ppGpp concentration (43), our model allows for sensitive calibration of cell division in response to changes in ppGpp levels.

Why it is advantageous for ppGpp to promote division remains unclear. With limited resources available to devote to cell wall synthesis, it may benefit *E. coli* to prioritize cell division over elongation. This could increase the number of daughter cells generated, which may strengthen the chances for some members of the population to survive until conditions improve. However, as all daughter cells would face the same unfavorable conditions, it is unclear whether this would actually lead to increased survival in the population. The activation of division during the stringent response contrasts with the SOS response, wherein division is inhibited until DNA damage can be repaired (73). More research is needed to uncover why it is advantageous for ppGpp to activate division.

This work raises several additional points for future inquiry. First, it is unclear whether DksA’s oppositional, ppGpp-dependent phenotypic effects are limited to cell size or if DksA also exerts ppGpp-dependent divergent effects on other phenotypes. Second, while our work indicates that the increased average length of ppGpp^0^ cells is due to division inhibition by DksA, it is still unclear why some ppGpp^0^ cells lengthen only slightly, while others form dramatic filaments. Future work focusing on single cells could determine why some ppGpp^0^ cells have slight division defects, while others seem to display a catastrophic failure to divide. Finally, although this work clearly demonstrates that ppGpp regulates division through DksA, the observation that *prelA* still caused a small (albeit non-significant) decrease in length in the *dksA::Kan* mutant leaves open the possibility that ppGpp may make additional minor contributions to cell size via non-transcriptional mechanism(s) (Fig. 1).

Altogether, our data indicate that DksA and ppGpp regulate divisome assembly through indirect modulation of FtsZ (Fig. 9). As the first protein recruited to the divisome, FtsZ represents an attractive target for the regulation of division. At the same time, the precise mechanism by which FtsZ is regulated by ppGpp is unclear. As previously stated, neither DksA nor ppGpp directly transcriptionally regulate *ftsZ* (11, 16, 45, 49, 50). Our data further suggest that these regulators also do not affect FtsZ levels, although our inability to detect a difference in FtsZ levels due to the *pftsZ* plasmid leaves open the possibility that small, undetectable changes in FtsZ levels do occur in these strains (Fig. S10 to S13). There are many well-characterized regulators of FtsZ, which control both the placement of the divisome and regulate division in response to stress (29–31, 74–77). Whether DksA and/or ppGpp affect these known regulatory pathways, or whether they modulate FtsZ through a novel mechanism, remains to be determined.

## MATERIALS AND METHODS

### Bacterial strains, plasmids, and growth conditions

Bacterial strains and plasmids used in this study are detailed in Table S1. All experiments were performed in the MG1655 background, referred to as “wild type.” Alleles of interest were moved between strains via P1 transduction; transductants were confirmed via PCR. *ftsZ84* RNAP_1−2−_ and *ftsZ84* RNAP_1+2+_ strains were generated using a *cat*-linked *ftsZ84* strain. A *cat* insertion in the *ftsZ*-linked *leuO* gene was generated by recombineering using plasmids pKD3 and pKD46 and primers listed in Table S1 (78). Plasmids *pftsZ* and *pftsQA* were generated from pBS58 (*pftsQAZ*) via Q5 mutagenesis (New England Biolabs, Ipswich, MA) using primers listed in Table S1. Plasmid *prelA-EV* was generated from *prelA* via *In Vivo* Assembly using primers listed in Table S1 (79). Primers were acquired from Integrated DNA Technologies (Coralville, IA).

Unless otherwise indicated, all chemicals, media components, and antibiotics were purchased from Sigma-Aldrich (St. Louis, MO). Experiments were performed in LB broth (1% tryptone, 1% NaCl, 0.5% yeast extract) or LBNS broth (1% tryptone, 0.5% yeast extract), as indicated. When indicated, cultures were supplemented with 0.2% glucose or IPTG (see “Fluorescence imaging of division proteins” for concentrations). When selection was necessary, cultures were supplemented with 50 µg/mL kanamycin (Kan), 30 µg/mL chloramphenicol (Cm), 12.5 µg/mL tetracycline (Tet), 100 µg/mL ampicillin (Amp), or 100 µg/mL spectinomycin (Spec).

### Image acquisition

Phase contrast and fluorescence imaging was performed using samples on 1% agarose/PBS pads with an Olympus BX51 microscope equipped with a 100× Plan N (N.A. = 1.25) Ph3 objective (Olympus), X-Cite 120 LED light source (Lumen Dynamics), and an OrcaERG CCD camera (Hammamatsu Photonics) or a Nikon TiE inverted microscope equipped with a 100× Plan N (N.A. = 1.25) objective (Nikon), SOLA SE Light Engine (Lumencor), heated control chamber (OKO Labs), and ORCA-Flash4.0 sCMOS camera (Hammamatsu Photonics). Filter sets for fluorescence were bought from Chroma Technology Corporation. Nikon Elements software (Nikon Instruments) was used for image capture.

### Cell size analysis

Cells were cultured from a single colony and grown to exponential phase (OD_600_ ≥ ~0.1). Cultures were then back diluted into fresh media to an OD_600_ of 0.005 and grown to exponential phase (OD_600_ = 0.1–0.2). Cultures were monitored during growth to determine the growth rate (see “Growth rate determination”). Cultures were fixed by adding 500 µL culture to 20 µL of 1 M sodium phosphate (pH 7.4) and 100 µL of fixative (16% paraformaldehyde and 8% glutaraldehyde). Samples were incubated at room temperature for 15 min and ice for 30 min. Samples were then stored at 4°C for up to overnight before being pelleted, washed three times in 1 mL PBS, and resuspended in glucose-tris-EDTA (GTE). Samples were stored at 4°C and used for imaging within 1 week of fixation. Cell length and width were measured from phase contrast images using the FIJI plugin MicrobeJ (62).

### Heat sensitivity experiments

Strains were grown in LB or LB-glucose (supplemented with 100 µg/mL Amp as appropriate) at 30°C until mid-log phase (OD_600_ = ~0.2–0.6). Cells were pelleted, washed once in LBNS, and resuspended in LBNS to an OD_600_ of 1.0. Cells were serially diluted from 10^−1^ to 10^−6^ in LBNS and 5 µL of each dilution was spot plated on LB (control) and LBNS plates. Plates were incubated at 30°C (LB, LBNS), 37°C, and 42°C (LBNS only) for 20 h prior to imaging.

### Fluorescence imaging of division proteins

Strains producing GFP fusion proteins were grown, sampled, and fixed as in “Cell size analysis.” Strains were grown in the following concentrations of IPTG: *gfp-ftsZ*—1 mM; *gfp-ftsA* and *gfp-ftsL*—100 µM; *gfp-ftsI*—2.5 µM; and *gfp-ftsN*—5 µM, as done previously (33). Fixed samples were imaged within 48 h of fixation. Phase contrast and fluorescence images were acquired on a Nikon TiE inverted microscope. Cell lengths were measured as for “Cell size analysis.” GFP rings were manually counted in FIJI. *L*/*R* was calculated using the following formula: $\frac{L}{R}=\frac{\Sigma \left(cell\;lengths\right)}{\Sigma \left(rings\right)}$.

### Growth rate determination

Growth rates were determined from growth curves of cultures used for fixation (see “Cell size analysis”). OD_600_ was measured every ~30 min, and values were plotted. OD_600_ values within early exponential phase (up to an OD_600_ of 0.2–0.3) were used to calculate the growth rate using the Doubling Time Cell Calculator++ (https://doubling-time.com/compute_more.php).

### Western blotting

Strains were grown in LB glucose (wild type, ppGpp^0^, *dksA::Kan*, and ppGpp^0^ *dksA::Kan*), LB Amp (*prelA’* and *prelA*), LB Spec (*pftsQAZ*; Fig. S13), or LB glucose Spec (*pftsZ* and *pftsQAZ*; Fig. S10) to mid-log phase (OD_600_ ~0.3–0.5). Samples were back diluted to an OD_600_ of 0.01 into the same type of media and grown to an OD_600_ of ~0.4. Samples were pelleted and stored at −80°C. Samples were thawed on ice and resuspended in LB to an OD_600_ of 1.6 before being diluted in 4× Laemmli buffer (Bio-Rad, Hercules, CA) with 2-mercaptoethanol and boiled for 10 min. Ten microliters of each sample was electrophoresed on a 10% Mini-Protean Precast gel (Bio-Rad) in 25 mM Tris base, 192 mM glycine, and 0.1% SDS at 200 V. Proteins were transferred to a PVDF membrane at 25 V for ~1.5 h using an XCell II Blot Module (Invitrogen Life Technologies, Carlsbad, CA) in 12.5 mM Tris base, 95 mM glycine, and 20% methanol. Following transfer, membranes were washed for 10 min in PBS. Membranes were agitated in Ponceau stain (G-Biosciences, St. Louis, MO) for 5 min to stain total protein, followed by three washes in PBS 5% acetic acid. Total protein was imaged using an Epson Perfection V600 Photo scanner. Membranes were destained with 0.1 M sodium hydroxide, washed for 5 min in PBS, and blocked for 1 h in 5% milk in PBS. Membranes were incubated overnight in 1:5,000 rabbit α-FtsZ (Cocalico Biologicals Inc., Stevens, PA) in PBS. Membranes were washed three times for 5 min in PBS 0.05% Tween, followed by a 1-h incubation in 1:5,000 goat α-rabbit HRP-conjugated antibody (Thermo Fisher Scientific, Waltham, MA) in PBS. Membranes were washed three more times in PBS Tween, rinsed twice in PBS, and imaged using Clarity Western ECL Substrate (Bio-Rad) on a LiCor Odyssey imager. Quantitation was determined in FIJI and normalized to Ponceau staining as a total protein loading control (80).

### ppGpp^0^ suppressor tests

ppGpp^0^ strains readily accumulate suppressor mutations in RNAP genes (81). For all experiments using ppGpp^0^ and its derivatives, cultures were tested for the presence of suppressors at the same time that experimental samples were acquired. Five hundred microliters of culture was pelleted, and cells were washed once with 1 mL AB media (10 mM ammonium sulfate, 40 mM disodium phosphate, 20 mM monopotassium phosphate, 50 mM sodium chloride, 100 µM calcium chloride, 1 mM magnesium chloride, and 3 µM ferric chloride). Cells were resuspended in AB media to an OD_600_ ~1 and serially diluted from 10^−1^ to 10^−6^. Five microliters of each dilution was spot plated on LB and AB 0.2% glucose. Plates were incubated at 37°C overnight (LB) or for up to 48 h (AB-glucose). As ppGpp^0^ is unable to grow on minimal media without amino acids, the frequency of suppressors was determined by dividing the CFU on AB-glucose by the CFU on LB. Data were only included from experiments where the frequency of suppressors was <10%.

### Quantification and statistical analyses

All experiments were performed with at least three biological replicates. Microscopy experiments were performed using at least 200 cells per replicate, unless otherwise indicated in figure legends. Statistical tests were performed as indicated in figure legends using GraphPad Prism 7.

## SUPPLEMENTAL MATERIAL

Supplemental Figures mbio.02425-23-s0001.docx Figures S1-S13.

Table S1 mbio.02425-23-s0002.docx Bacterial strains, plasmids, and primers.

ASM does not own the copyrights to Supplemental Material that may be linked to, or accessed through, an article. The authors have granted ASM a non-exclusive, world-wide license to publish the Supplemental Material files. Please contact the corresponding author directly for reuse.
