# Supplementary material for: The transcription factor DksA exerts opposing effects on cell division depending on the presence of ppGpp
Source: mBio. 2023 Oct 26;14(6):e02425-23. doi: 10.1128/mbio.02425-23 (PMC10746185; doi:10.1128/mbio.02425-23)
Supplement: Supplemental Figures — Figures S1-S13. [file mbio.02425-23-s0001.docx]

**Supplemental Figure S1.**


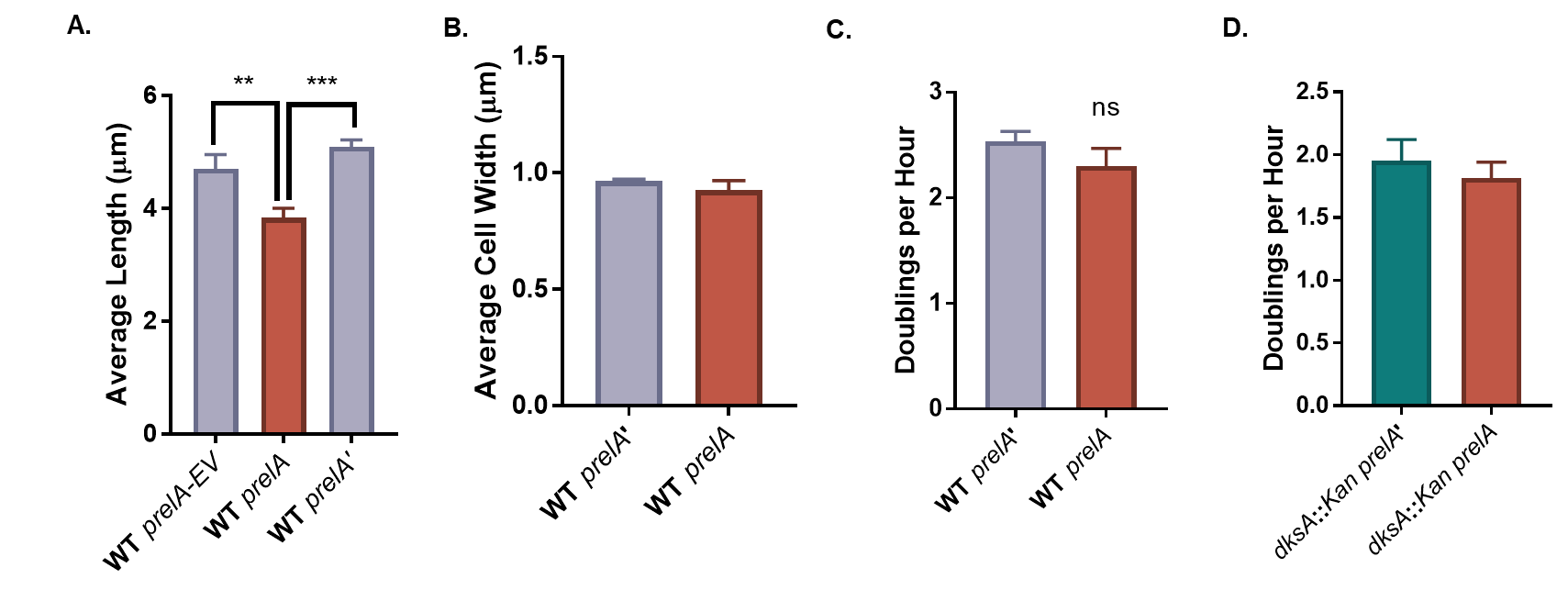


**Supplemental Figure S1. Cell lengths, widths, and growth rates of strains expressing different *relA* plasmids. A.** Average length of WT cells expressing *prelA*, *prelA’*, or the empty vector control *prelA-EV*. Data represent averages and SDs of three independent replicates (**, *P* ≤ 0.01; **, *P* ≤ 0.001 by one-way ANOVA with Tukey’s post-test). **B.** Average widths of cells expressing *prelA* and *prelA’*. Data represent averages and SDs of at least three independent replicates. Difference is not significant by two-tailed t-test. **B-C.** Population mass doubling times of cells expressing *prelA* in the wild-type (**B**) or *dksA*::*Kan* (**C**) background. Data represent averages and SDs of at least three independent replicates; differences are not significant by two-tailed t-test.

**Supplemental Figure S2.**


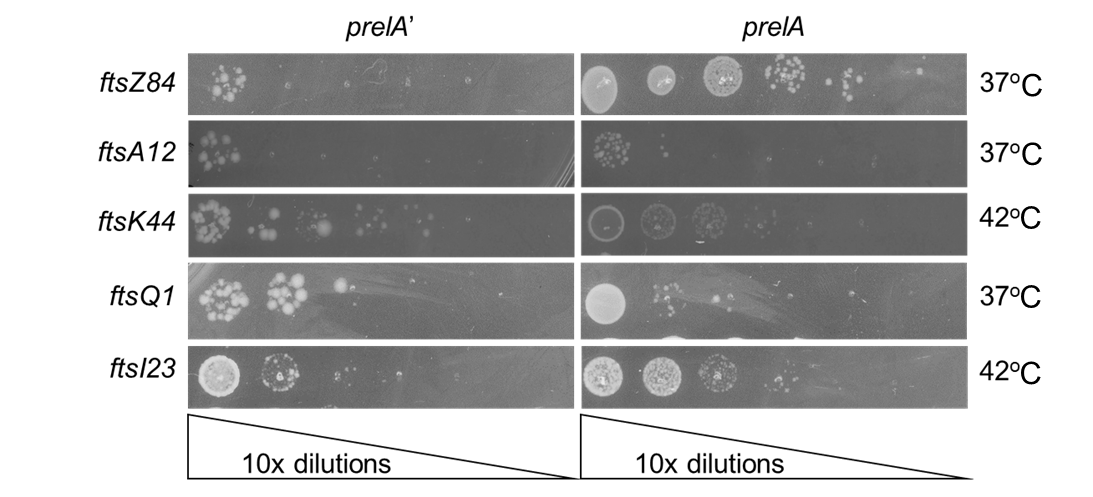


**Supplemental Figure S2. Effect of *relA* overexpression on conditional division mutants.** Effect of overexpression of *relA* on growth of heat-sensitive division mutants at restrictive temperatures. Data shown are representative images of three biological replicates.

**Supplemental Figure S3.**


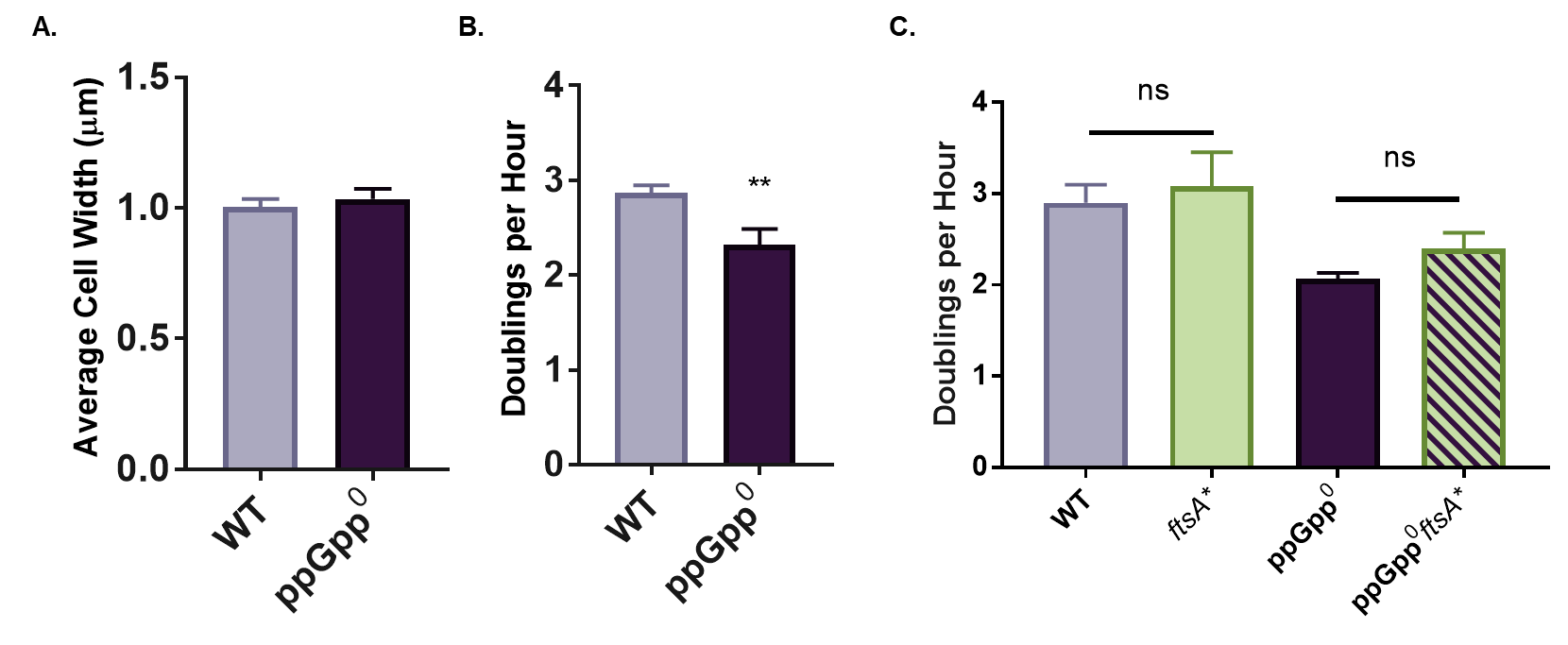


**Supplemental Figure S3. Cell widths and growth rates of ppGpp^0^. A.** Average widths of cells lacking ppGpp. Data represent averages and SDs of at least three independent replicates. Differences are not significant by two-tailed t-test. **B.** Growth rates of cells lacking ppGpp. Data represent averages and SDs of at least three independent replicates (**, *P* ≤ 0.01 by two-tailed t-test). **C.** Mass doubling times of ppGpp^0^ and ppGpp^0^ *ftsA** strains. Data represent averages and SDs of at least three independent replicates (ns, not significant by one-way ANOVA with Tukey’s multiple comparison test).

**Supplemental Figure S4.**


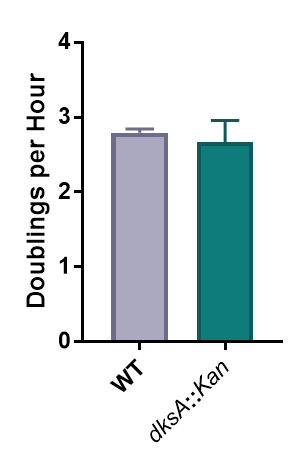


**Supplemental Figure S4. Growth rate of *dksA* mutant.** Data represent averages and SDs of three independent replicates. Differences are not significantly different by two-tailed t-test.

**Supplemental Figure S5.**

**
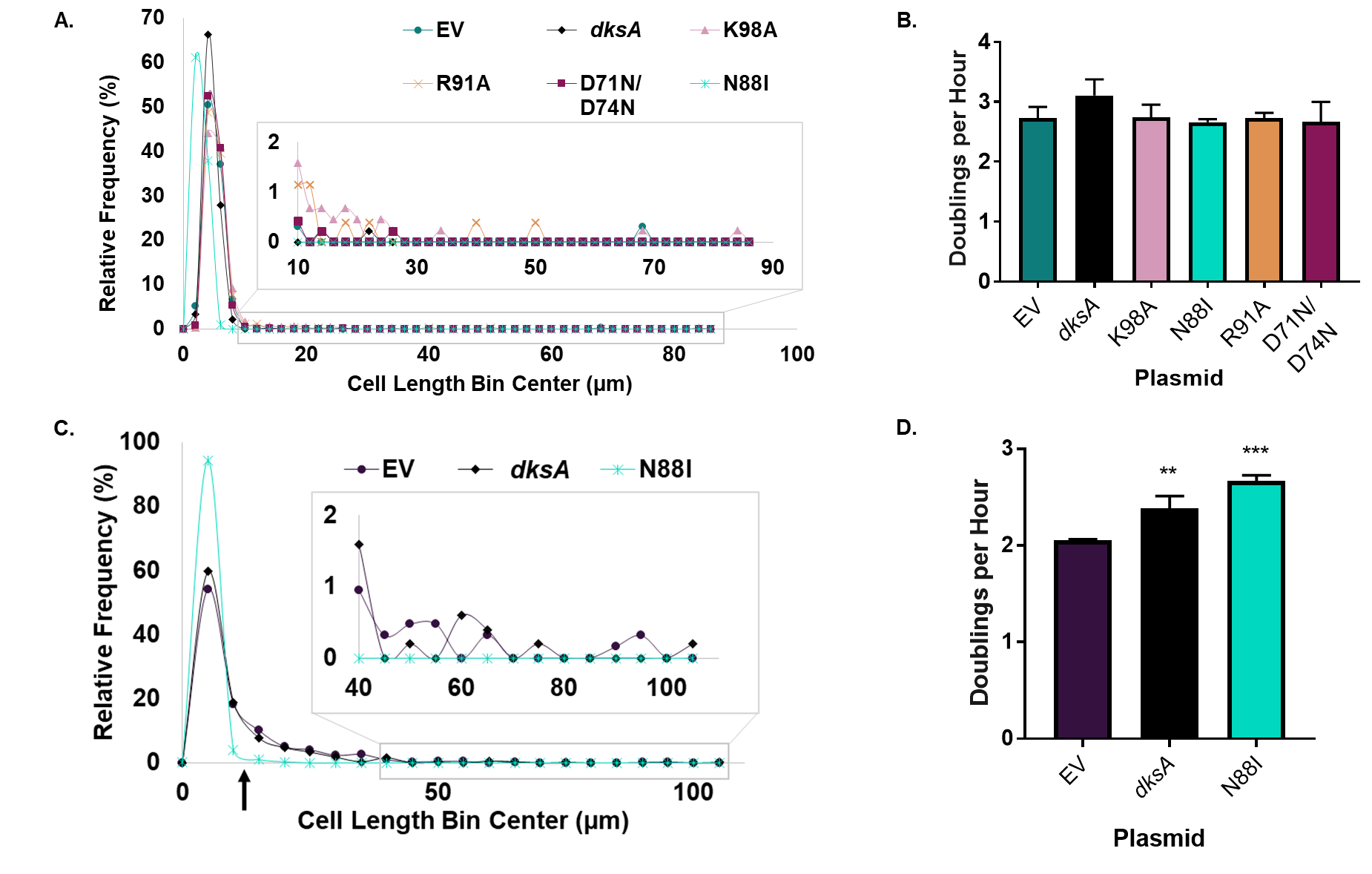
**

**Supplemental Figure S5. Growth rates and single cell sizes for *dksA*::*Kan* and ppGpp^0^ strains expressing different *dksA* alleles. A.** Frequency distribution of individual cell lengths for *dksA*::*Kan* complemented with different *dksA* alleles*.* N > 300 cells from a single representative experiment (bin width = 2 µm). **B.** Expression of different *dksA* alleles from a plasmid has no effect on growth rates of a *dksA*::*Kan* mutant. Data represent averages and SDs of three biological replicates (differences relative to EV are not significant by one-way ANOVA with Dunnett’s post-test). **C.** Frequency distribution of individual cell sizes show that *dksA*_N88I_ reduces length and largely eliminates filamentation in ppGpp^0^. N > 500 cells from a single representative experiment (bin width = 5 µm). The black arrow indicates the approximate location of the cut-off for filamentous cells (12.4 µm, 3x wild-type cell length in Fig. 3C). **D.** Expression of *dksA* and *dksA*_N88I_ increases the growth rate of ppGpp^0^ cells. Data represent averages and SDs of three biological replicates (**, *P* ≤ 0.01; ***, *P* ≤ 0.001 relative to EV by one-way ANOVA with Dunnett’s post-test).

**Supplemental Figure S6.**


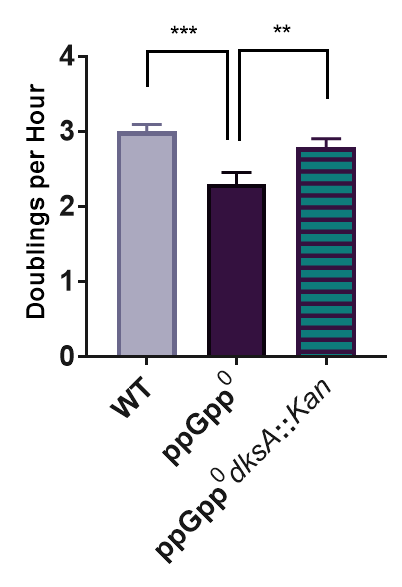


**Supplemental Figure S6. Deletion of *dksA* increases growth rate of ppGpp^0^.** Data represent averages and SDs of three biological replicates (**, *P* ≤ 0.01; ***, *P* ≤ 0.001 relative to ppGpp^0^ by one-way ANOVA with Dunnett’s post-test).

**Supplemental Figure S7.**


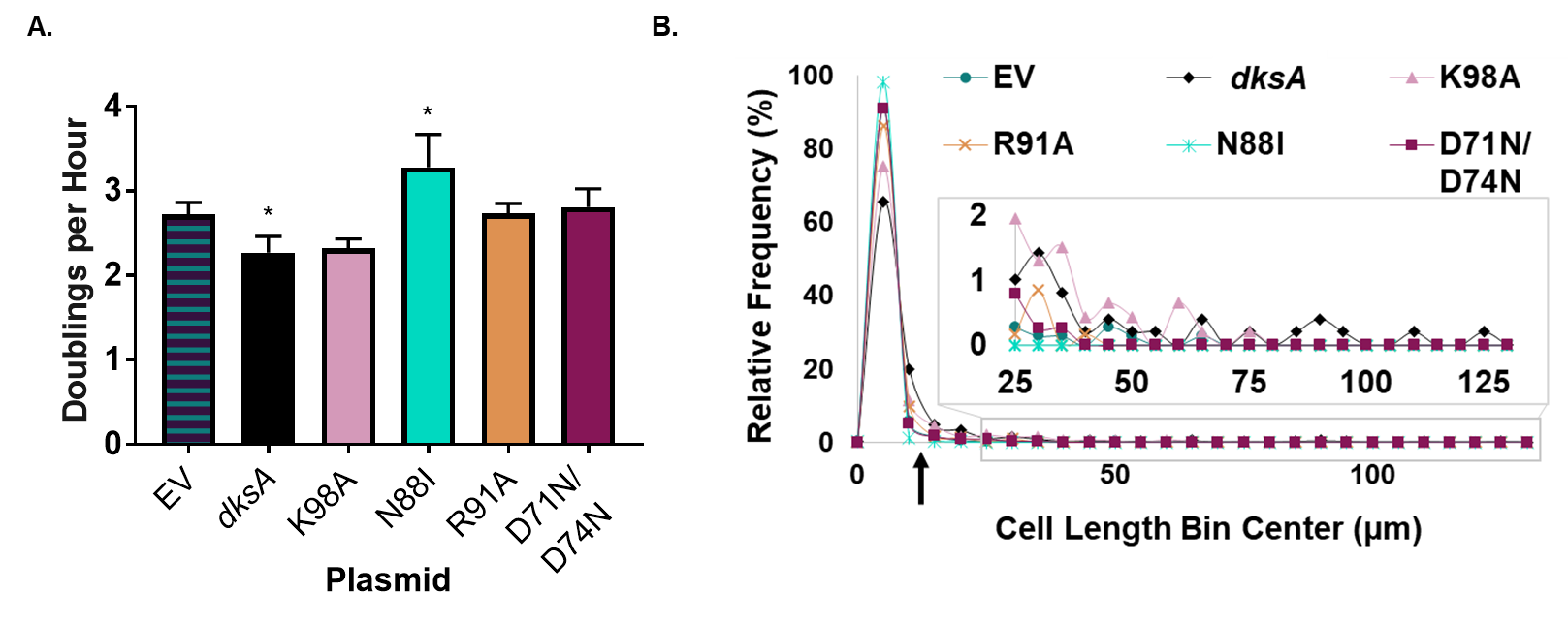


**Supplemental Figure S7. Growth rates and individual cell sizes of ppGpp^0^ *dksA*::*Kan* expressing different *dksA* alleles. A.** Growth rates of ppGpp^0^ *dksA*::*Kan* mutants complemented with different *dksA* alleles. Complementation with wild-type *dksA* reduces the growth rate, while complementation with *dksA*_N88I_ leads to increased growth rate. Data represent averages and SDs of three independent replicates (*, *P* ≤ 0.05 relative to EV by one-way ANOVA with Dunnett’s post-test). **B.** Frequency distribution of individual cell lengths for ppGpp^0^ *dksA*::*Kan* mutants complemented with different *dksA* alleles. N > 300 cells from a single representative experiment (bin width = 5 µm). The arrow indicates the approximate location of the cut-off for filamentous cells (12.2 µm, 3x wild-type length from Fig. 7A).

**Supplemental Figure S8.**


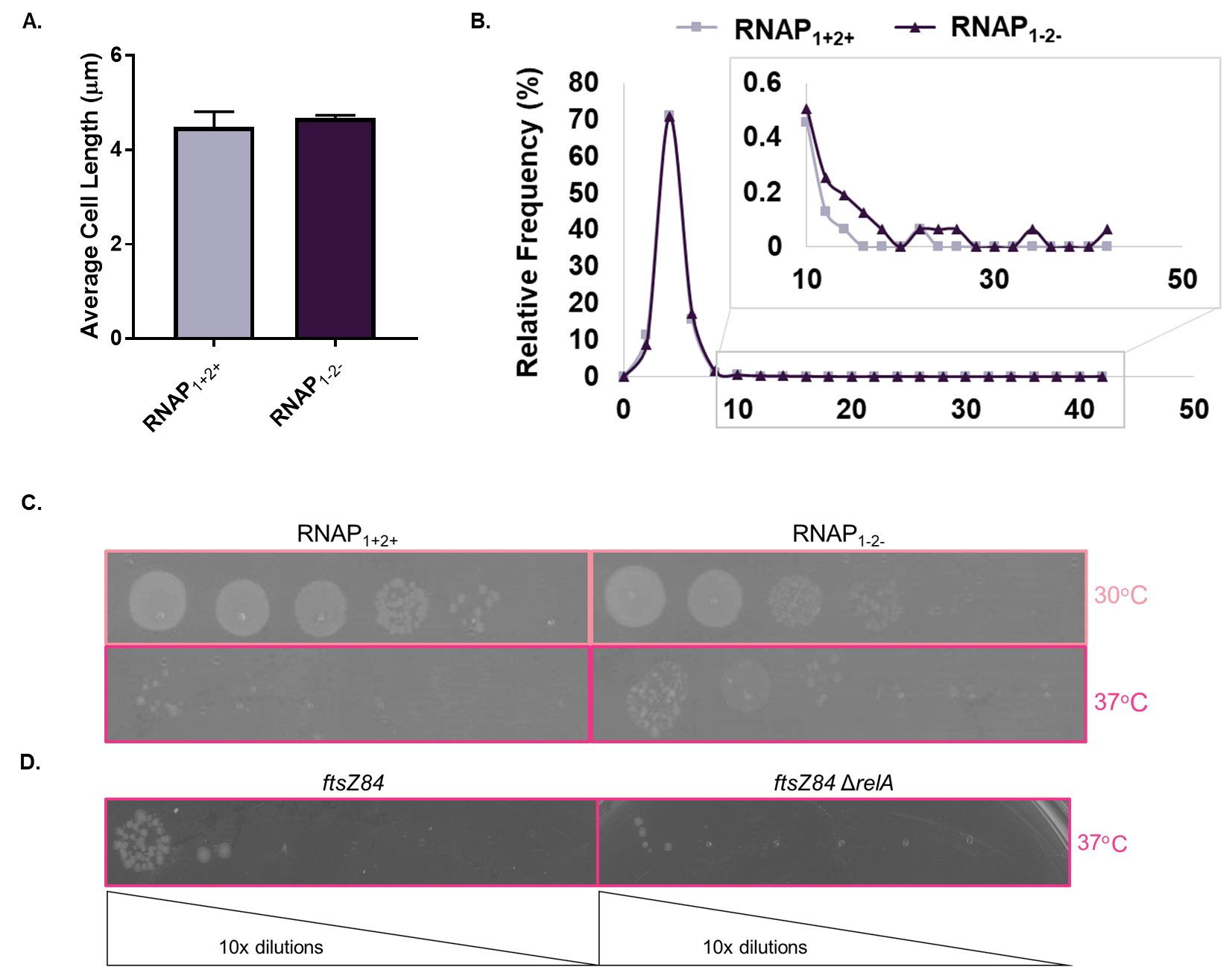


**Supplemental Figure S8. Mutations blocking ppGpp binding to RNAP do not affect cell length. A.** There is no significant difference in cell length between an RNAP_1-2-_ mutant and an RNAP_1+2+_ control by two-tailed t-test. Data represent averages and SDs of three independent replicates. **B.** Distribution of individual cell lengths reveal that RNAP_1-2­-_ cells have very similar lengths to RNAP_1+2+_ cells. N > 1500 cells from a single representative experiment (bin width = 2 µm). **C.** An RNAP_1-2­-_ *ftsZ84* exhibits a slight decrease in CFUs at 30^o^C and an increase in CFUs at 37^o^C compared to an RNAP_1+2+_ *ftsZ84* control. A representative image from three biological replicates is shown. **D.** Deletion of *relA* does not affect growth of *ftsZ84* at the non-permissive temperature of 37^o^C. A representative image from three biological replicates is shown.

**Supplemental Figure S9.**

**
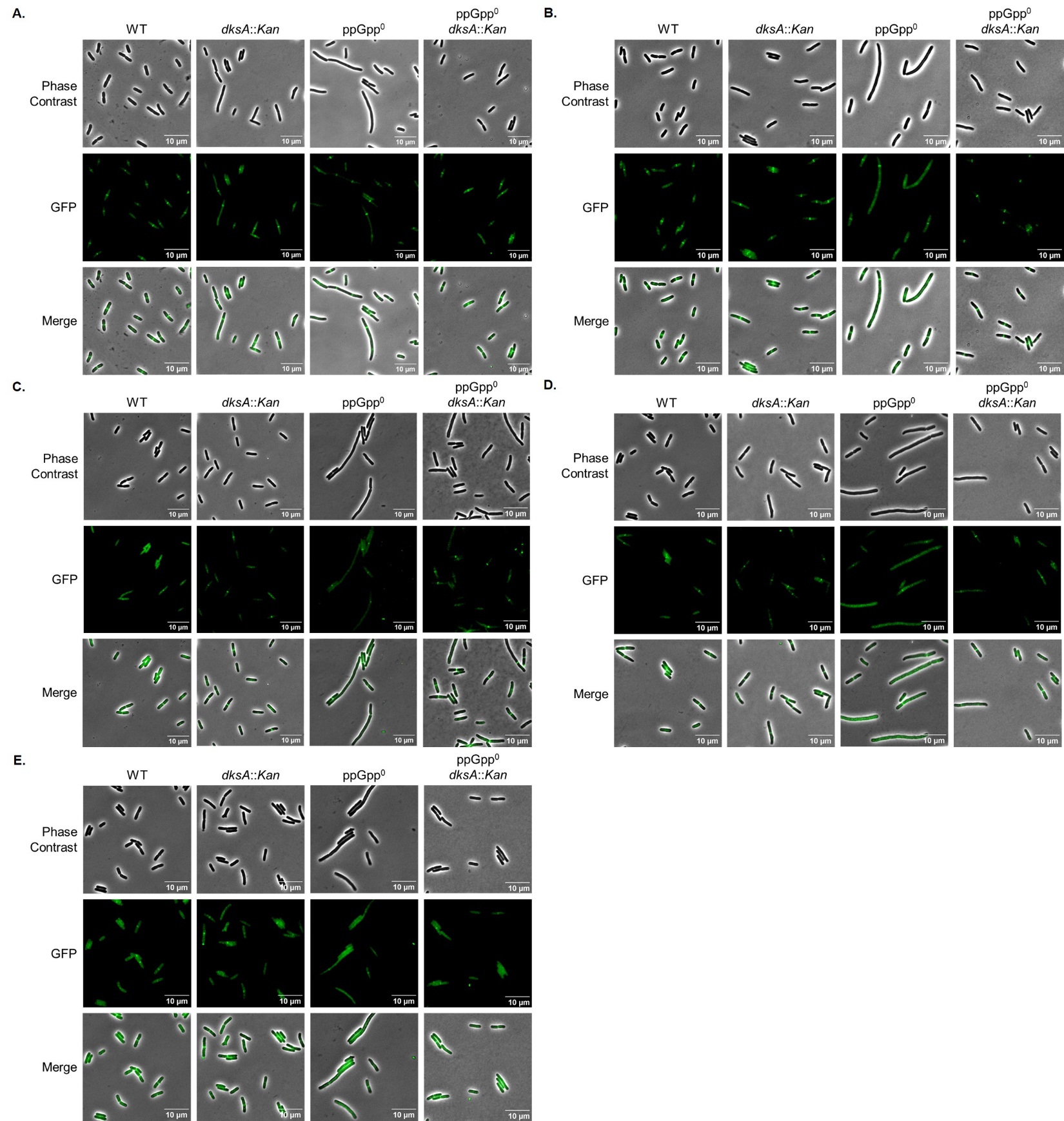
**

**Supplemental Figure S9. ppGpp^0^ cells exhibit decreased recruitment of division proteins.** Phase contrast and fluorescence images of cells expressing GFP-FtsZ (**A**), GFP-FtsA (**B**), GFP-FtsL (**C**), GFP-FtsI (**D**), or GFP-FtsN (**E**) are shown. Images are representative of at least three biological replicates.

**Supplemental Figure S10.**

**
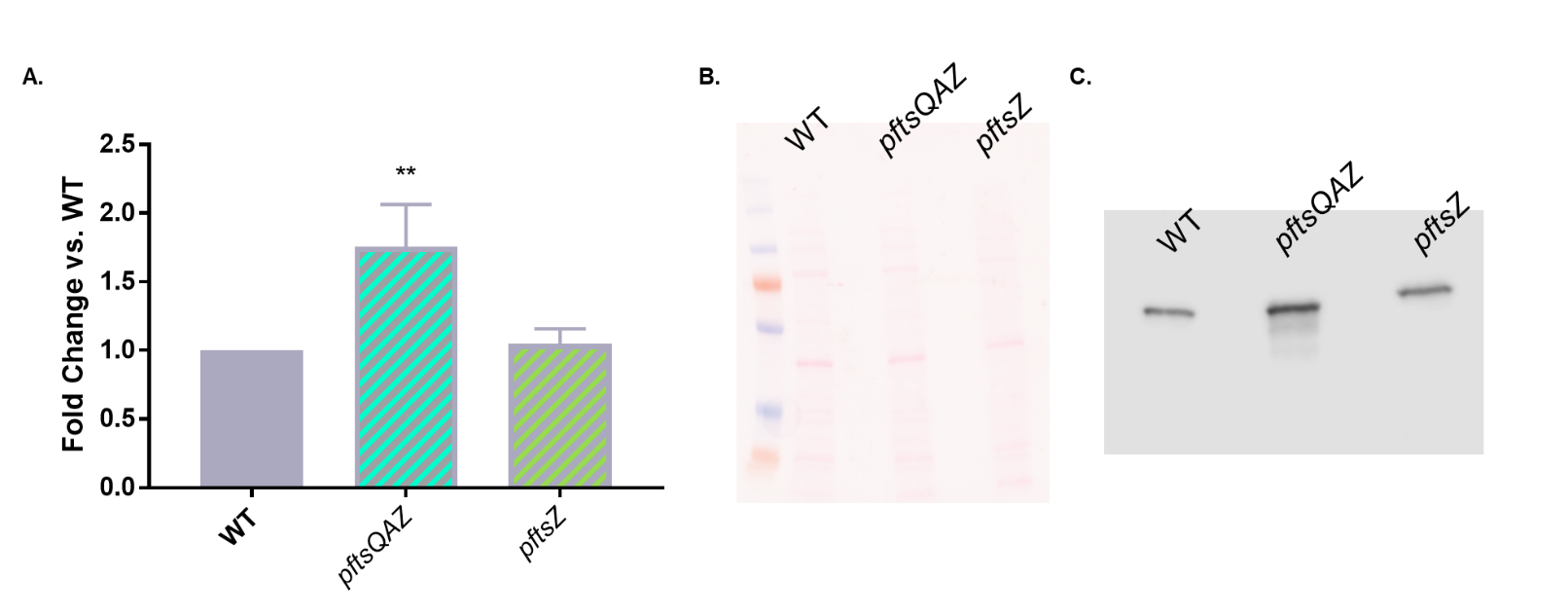
**

**Supplemental Figure S10. FtsZ levels in WT cells encoding *pftsQAZ* and *pftsZ*. A.** Quantification of FtsZ levels in WT cells expressing *pftsQAZ* and *pftsZ* relative to a WT control. Data represent averages and SDs of three independent replicates (**, *P* ≤ 0.01 by one-way ANOVA with Dunnett’s post-test). **B, C.** A representative blot with Ponceau staining for total protein (**B**) and FtsZ immunostaining (**C**) is shown.

**Supplemental Figure S11.**


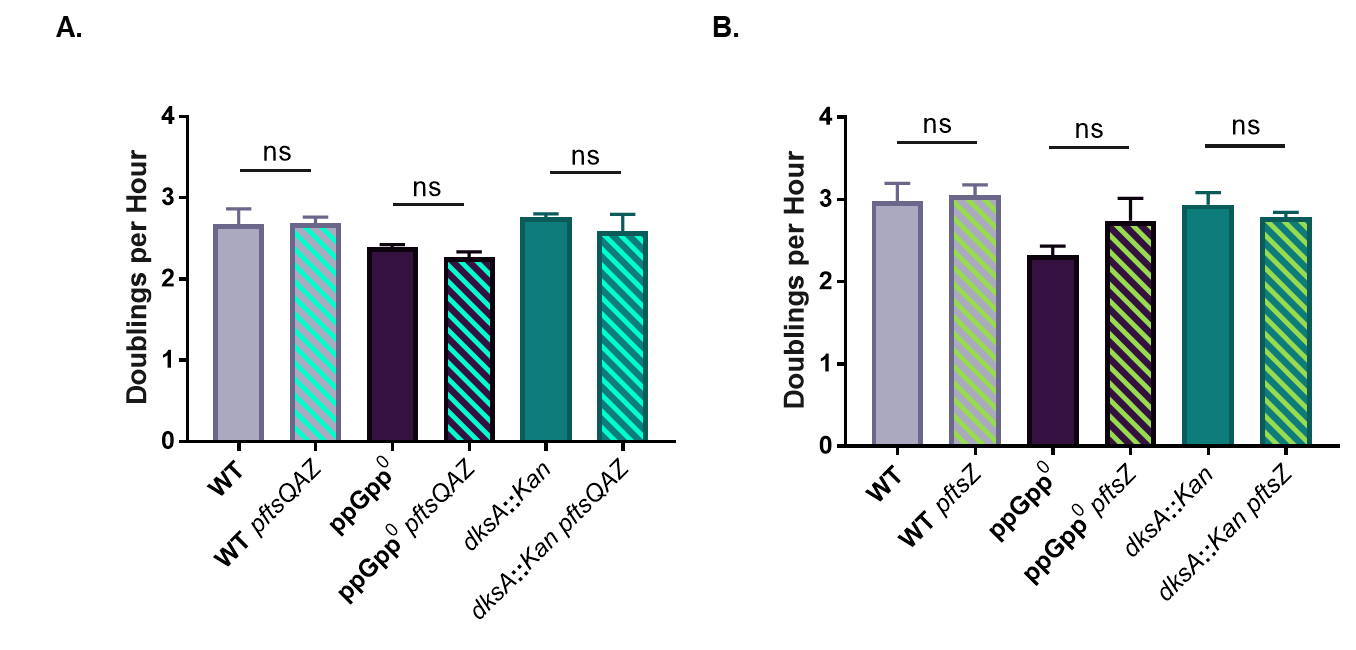


**Supplemental Figure S11. Ectopic expression of *ftsZ* does not lead to changes in growth rate.** No significant differences in growth rates were observed for strains overexpressing *ftsQAZ* (**A**) or *ftsZ* (**B**). Data represent averages and SDs of three biological replicates (ns, not significant by two-tailed t-test).

**Supplemental Figure S12.**


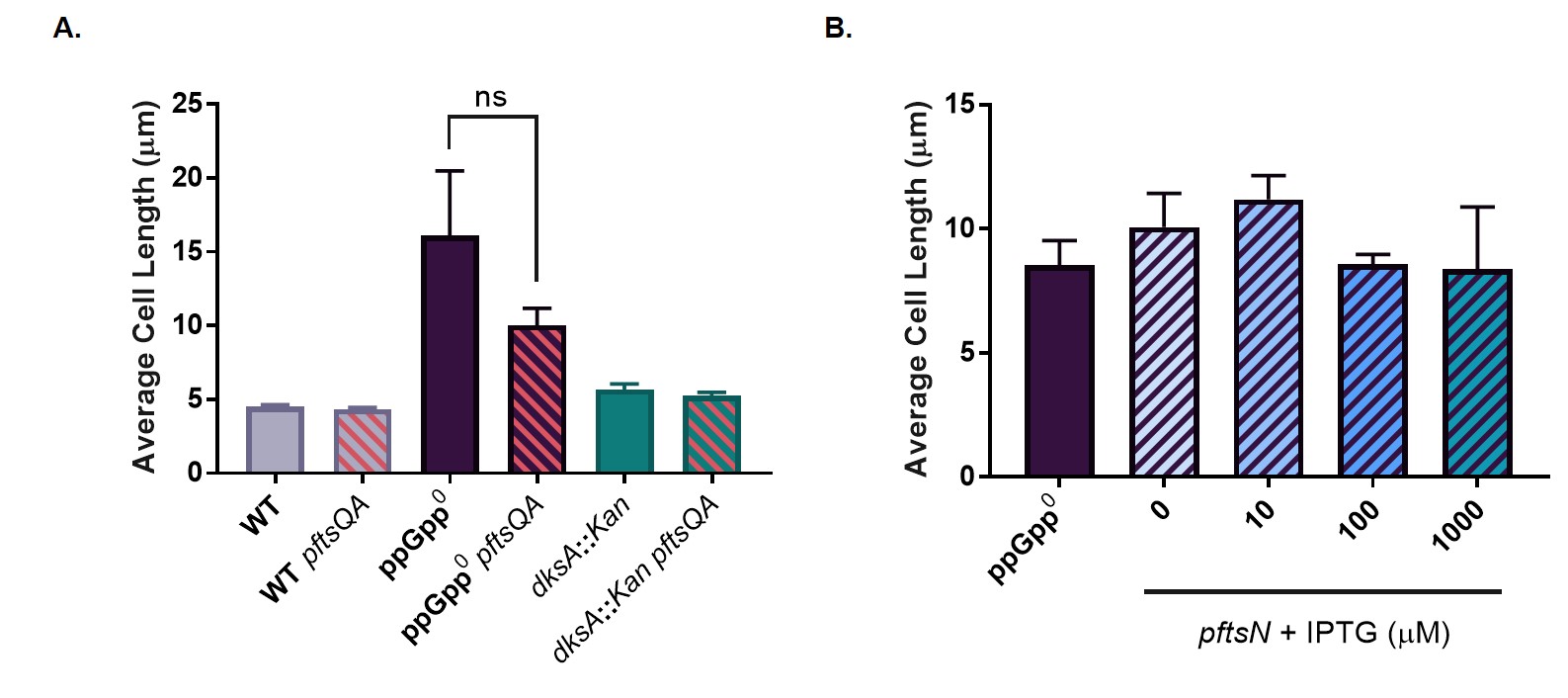


**Supplemental Figure S12. *pftsQA* and *pftsN* do not reduce the length of ppGpp^0^ cells.** **A.** Overexpression of *ftsQA* does not lead to differences in cell length in any background. Data shown represent averages and SDs from three biological replicates. Differences are not significant by two-tailed t-test. **B.** Overexpression of *gfp-ftsN* from an IPTG-inducible plasmid has no effect on length of ppGpp^0^ cells. Data shown represent averages and SDs from three biological replicates; differences are not significant by one-way ANOVA with Dunnett’s post-test relative to ppGpp^0^_._

**Supplemental Figure S13.**


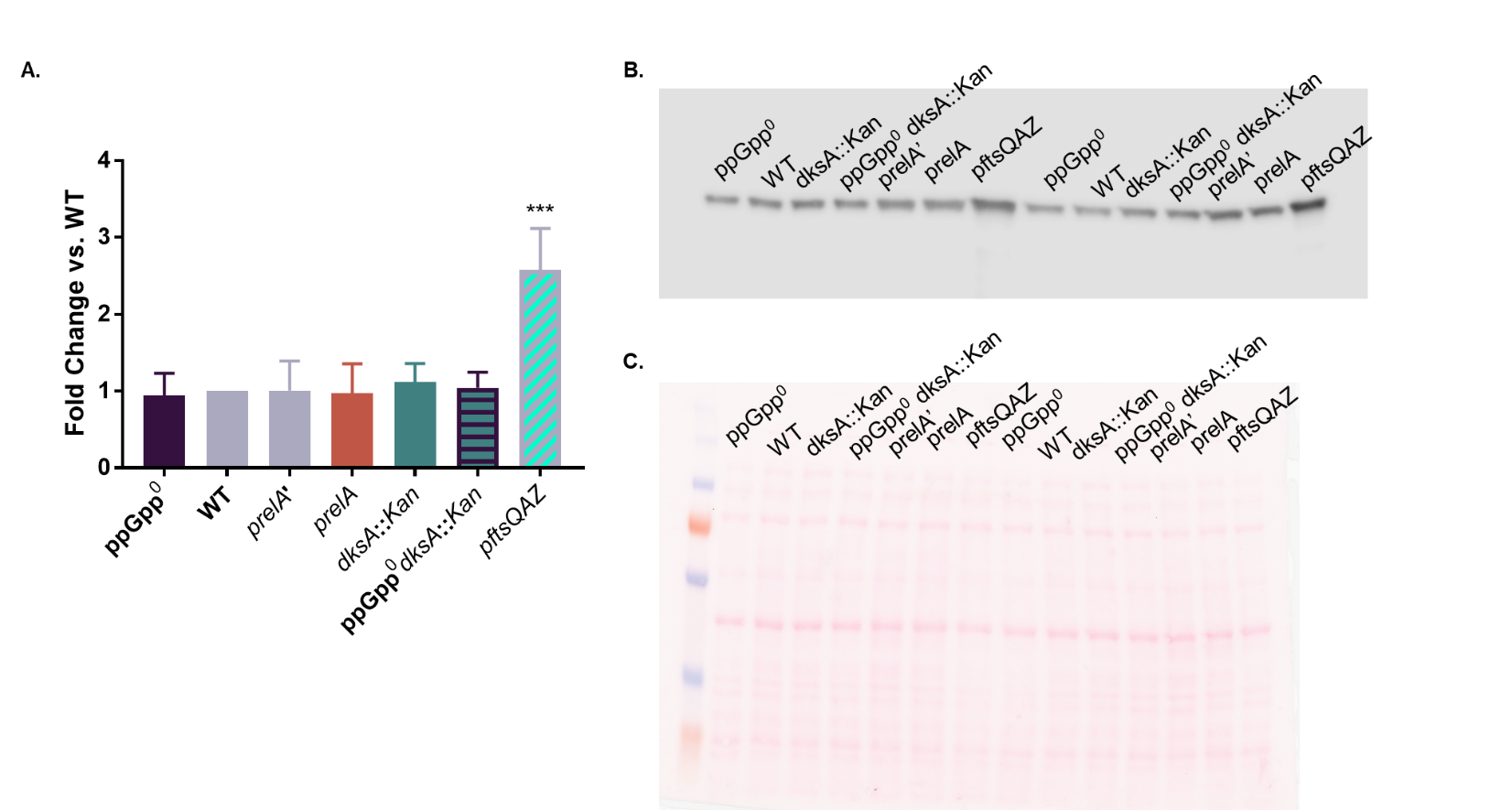


**Supplemental Figure S13. DksA and ppGpp do not affect FtsZ levels. A.** Strains with varying levels of *dksA* or ppGpp have no change in FtsZ levels compared to wild-type. FtsZ concentrations were normalized to total protein and to wild-type. Data represent averages and SDs of three independent replicates (**, *P* ≤ 0.01 relative to wild-type by one-way ANOVA with Dunnett’s post-test). **B.** A representative FtsZ immunoblot is shown. **C.** A representative Ponceau stain for total protein is shown. Samples in **B** and **C** were electrophoresed in technical duplicate.
